# Dynamic Surface Reconstruction of Amphoteric Metal (Zn, Al) Doped Cu_2_O for Efficient Electrochemical CO_2_ Reduction to C_2+_ Products

**DOI:** 10.1002/advs.202303726

**Published:** 2023-08-02

**Authors:** Yufei Jia, Yunxuan Ding, Tao Song, Yunlong Xu, Yaqing Li, Lele Duan, Fei Li, Licheng Sun, Ke Fan

**Affiliations:** ^1^ State Key Laboratory of Fine Chemicals Institute of Artificial Photosynthesis DUT‐KTH Joint Education and Research Centre on Molecular Devices Institute for Energy Science and Technology Dalian University of Technology Dalian 116024 P. R. China; ^2^ Center of Artificial Photosynthesis for Solar Fuels, Department of Chemistry School of Science Westlake University Hangzhou 310024 P. R. China; ^3^ Department of Chemistry and Shenzhen Grubbs Institute Southern University of Science and Technology Shenzhen 518055 P. R. China

**Keywords:** amphoteric metal‐doped Cu_2_O, CO_2_ reduction, electrocatalysis, leaching, redeposition

## Abstract

The recognition of the surface reconstruction of the catalysts during electrochemical CO_2_ reduction (CO2RR) is essential for exploring and comprehending active sites. Although the superior performance of Cu–Zn bimetallic sites toward multicarbon C_2+_ products has been established, the dynamic surface reconstruction has not been fully understood. Herein, Zn‐doped Cu_2_O nano‐octahedrons are used to investigate the effect of the dynamic stability by the leaching and redeposition on CO2RR. Correlative characterizations confirm the Zn leaching from Zn‐doped Cu_2_O, which is redeposited at the surface of the catalysts, leading to dynamic stability and abundant Cu–Zn bimetallic sites at the surface. The reconstructed Zn‐doped Cu_2_O catalysts achieve a high Faradaic efficiency (FE) of C_2+_ products (77% at –1.1 V versus reversible hydrogen electrode (RHE)). Additionally, similar dynamic stability is also discovered in Al‐doped Cu_2_O for CO2RR, proving its universality in amphoteric metal‐doped catalysts. Mechanism analyses reveal that the OHC–CHO pathway can be the C–C coupling processes on bare Cu_2_O and Zn‐doped Cu_2_O, and the introduction of Zn to Cu can efficiently lower the energy barrier for CO2RR to C_2_H_4_. This research provides profound insight into unraveling surface dynamic reconstruction of amphoteric metal‐containing electrocatalysts and can guide rational design of the high‐performance electrocatalysts for CO2RR.

## Graphical abstract

Zn leaching from Zn‐doped Cu_2_O is redeposited at the surface of the catalysts in electrocatalytic CO_2_ reduction, leading to dynamic stability and abundant Cu–Zn bimetallic sites at the surface, which achieves a high Faradaic efficiency of C_2+_ products. Similar dynamic stability is also discovered in Al‐doped Cu_2_O for CO_2_ reduction, proving its universality in amphoteric metal‐doped catalysts.

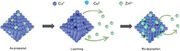

## 1 Introduction

The concentration of carbon dioxide (CO_2_) in the atmosphere of the Earth has increased from 280 ppm before the industrial revolution to the current level of 410 ppm, and is considered the primary contributor to the climate change. Conversion of CO_2_ to hydrocarbons and/or oxygenates is a promising strategy to alleviate CO_2_ emission. Electrochemical CO_2_ reduction (CO2RR), which converts CO_2_ into fuels and chemical feedstocks driven by renewable energies, has gained significant attention in the past decades.^[^ ^1^ ^]^ Among the various products generated from CO2RR, multicarbon (C_2+_) products (e.g., ethylene, acetate, ethanol, and n‐propanol) are highly desirable due to their high direct utilization values.^[^ ^2^ ^]^ However, the CO2RR performance of copper‐based electrocatalysts, the only family of electrocatalysts capable of efficiently generating C_2+_ products from CO2RR, has been greatly limited by competing hydrogen evolution reaction, C_1_ products (e.g., CO, formate, and CH_4_) and slow reaction kinetics.^[^ ^3^ ^]^

Various strategies have been employed to enhance the selectivity and activity of Cu‐based catalysts for CO_2_ reduction to C_2+_ products, including oxide derivation,^[^ ^4^ ^]^ crystal plane regulation,^[^ ^5^ ^]^ surface modification,^[^ ^6^ ^]^ and bimetallic site construction.^[^ ^7^ ^]^ Among these approaches, copper‐based bimetallic catalysts have demonstrated significant improvements in overpotentials, selectivity, and activity of C_2+_ products. Notably, Cu–Zn bimetallic sites can promote C–C coupling and thus enhance the selectivity of C_2+_ products for CO2RR remarkably.^[^ ^8^ ^]^ Moreover, it is described that Cu–Al electrocatalysts efficiently reduce CO_2_ to ethylene with a high Faradaic efficiency (FE), attributed to the formation of a favorable Cu coordination environment that enhances C–C dimerization.^[^ ^9^ ^]^ These reports indicate that doping amphoteric metals to Cu could improve the selectivity of C_2+_ products from CO2RR by providing the abundant Cu‐amphoteric metal sites for C–C dimerization.

Surface reconstructions of catalysts during electrocatalytic reactions (e.g., water oxidation and hydrogen evolution reactions) are ubiquitous and crucial for understanding the real active sites.^[^ ^10^ ^]^ The process of component leaching and redeposition, which can cause surface reconstructions, has not received adequate attention. For instance, recently, the concomitant leaching and redeposition of the Fe active sites in NiFe‐layered double hydroxides was demonstrated after manipulating both electrode and electrolyte components for electrocatalytic water oxidation,^[^ ^11^ ^]^ highlighting the effect of dynamic surface stability on the structure–catalytic activity relationship. Nevertheless, such dynamic stability formed by component leaching and redeposition in electrocatalytic CO2RR has been seldom reported. Considering the predominantly used alkaline electrolyte and the higher local pH at the electrode surface during CO2RR operation,^[^ ^12^ ^]^ the dissolution of the aforementioned amphoteric metals of Zn/Al or amphoteric oxides of ZnO/Al_2_O_3_ doped in Cu/Cu_2_O can be reasonably anticipated,^[^ ^13^ ^]^ and the underlying corresponding redeposition of these amphoteric metals/oxides could lead to dynamic stability at the surface, which is important for mechanism research but often overlooked in literature.

Herein, we employ Zn‐doped Cu_2_O nano‐octahedrons to investigate the dynamic surface reconstruction of catalysts under CO2RR. The leaching and redeposition of Zn in CO2RR form a dynamic stability at the surface of Cu_2_O through the co‐effect of the strong alkaline electrolyte and applied negative potentials. Meanwhile, the ratio of active Cu^0^ is increased with the Zn‐rich surface of the dynamically reconstructed catalysts. The presence of Zn in Cu_2_O can stabilize the Cu^0^ and provides abundant Cu–Zn sites near the surface of the catalysts through the leaching and redeposition mechanism, which further improves the selectivity of C_2+_ products for CO2RR. Consequently, the optimized catalyst exhibits a FE of 77% for C_2+_ products at **–**1.1 V versus reversible hydrogen electrode (RHE, all potentials mentioned below are versus RHE, unless noted otherwise). Additionally, the behavior of another amphoteric metal, Al, has also been verified to show similar dynamic leaching and redeposition in Al‐doped Cu_2_O catalysts during CO2RR, indicating the ubiquity of the dynamic stability of the amphoteric metal‐doped electrocatalysts for CO2RR. In situ attenuated total reflection Fourier‐transform infrared spectroscopy (ATR–FTIR) results and density functional theory (DFT) calculations reveal that the reduction of CO_2_ to C_2_H_4_ occurs via the OHC–CHO pathway on Cu_2_O and Zn‐doped Cu_2_O, and the reaction free energy of the rate‐determining step (RDS) on Zn‐doped Cu_2_O is lower than that on bare Cu_2_O catalysts, which leads to an improvement in the performance of CO_2_ reduction to C_2_H_4_ formation. These findings provide insights into the surface reconstruction of amphoteric metal‐based electrocatalysts, and suggest avenues for the further development of high‐performance electrocatalysts for CO2RR.

## 2 Results and Discussion

**Figure 1:**
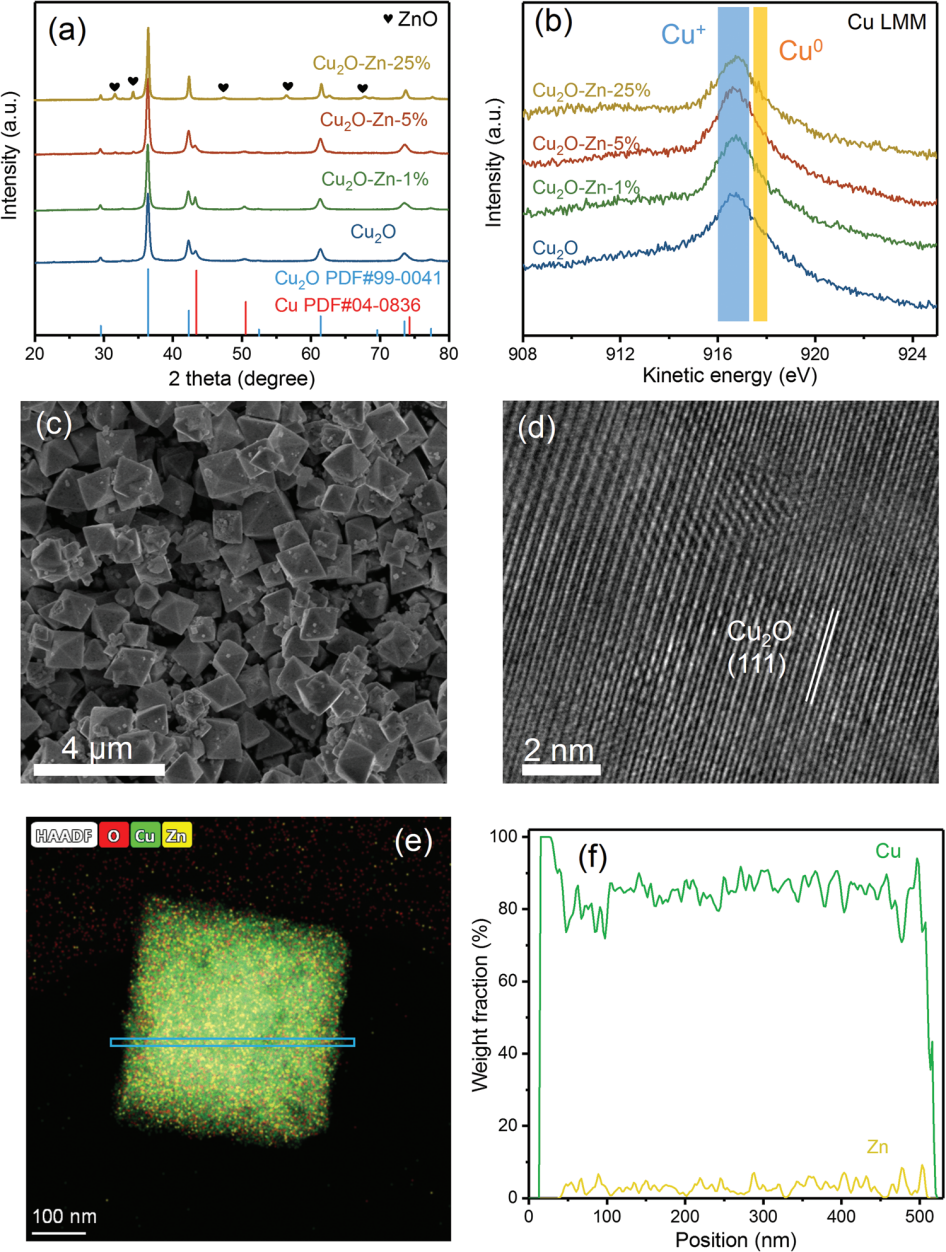
a) X‐ray diffraction (XRD) patterns and b) Auger electron spectroscopy (AES) spectra of Cu LMM of Cu_2_O, Cu_2_O–Zn‐1%, Cu_2_O–Zn‐5%, and Cu_2_O–Zn‐25%. c) Scanning electron microscopy (SEM) image, d) high‐resolution transmission electron microscopy (HRTEM) image, e) energy‐dispersive X‐ray spectroscopy (EDS) mapping image, and f) line profile of precursor Cu_2_O–Zn‐5%.

Zn‐doped Cu_2_O nano‐octahedrons precursor were prepared via low‐temperature chemical synthesis.^[^ ^14^ ^]^ To optimized the catalysts, the Zn content in the total amount of metals (Zn + Cu) was varied to 1 at%, 5 at% and 25 at% and investigated as Cu_2_O–Zn‐1%, Cu_2_O–Zn‐5% and Cu_2_O–Zn‐25% respectively. The X‐ray diffraction (XRD) patterns of the bare precursor Cu_2_O and precursor Zn‐doped Cu_2_O catalysts are presented in **Figure** **1**a. All the as‐prepared samples exhibit the strong diffraction peaks indexed to Cu_2_O. The Raman spectra of precursor Zn‐doped Cu_2_O (Figure S1, Supporting Information) also confirm the presence of Cu_2_O peaks at Raman shift values of 97, 218, and 528 cm**^–^** ^1^.^[^ ^15^ ^]^ In addition, the XRD patterns in Figure 1a show the presence of metallic *fcc* Cu^0^ phase peaks, which can be attributed to the strong reduction ability of the hydrazine hydrate used in the preparation process. However, the phase of Zn species is not evident in precursor Cu_2_O–Zn‐1% due to the low ratio of Zn or their small size. The phase of ZnO with zincite structure starts to appear (the peaks at 31.7°, 34.4°, and 47.5°) when the doping ratio of Zn increases to 5 and 25 at%. The control sample was also prepared by following the same synthesis protocol without the Cu precursor, and its XRD pattern (Figure S2, Supporting Information) and Raman spectrum (Figure S3, Supporting Information) also confirm the presence of the zincite ZnO phase,^[^ ^16^ ^]^ further suggesting that Zn exists in the form of ZnO in the precursor Cu_2_O–Zn samples from another perspective.

Further investigation on the chemical states of the samples was conducted through X‐ray photoelectron spectroscopy (XPS) and Auger electron spectroscopy (AES). The Cu 2p spectra of the four catalysts, depicted in Figures S4 (Supporting Information), display the strong peaks ascribed to Cu^0^ or Cu^+^ (933.0 eV) with the small peaks of Cu^2+^ (933.5 eV). In addition, Cu LMM spectra (Figure 1b) further reveal that the cuprous Cu^+^ (916.8 eV) is dominant in all the as‐prepared catalysts, with only a small amount of metallic Cu^0^ (918.6 eV) present.^[^ ^17^ ^]^ Based on the Zn 2p spectra (Figure S4c, Supporting Information), the state of Zn is confirmed to be Zn^2+^, consistent with the above XRD and Raman results.^[^ ^18^ ^]^

The typical octahedron structures of precursor Cu_2_O, Cu_2_O–Zn‐1%, and Cu_2_O–Zn‐5% are observed by scanning electron microscopy (SEM, Figure 1c, Figures S5 and S6, Supporting Information). The absence of isolated ZnO nanostructures can be confirmed in the high‐resolution transmission electron microscopy (HRTEM), indicating highly dispersed Zn atoms in precursor Cu_2_O–Zn‐1% and precursor Cu_2_O–Zn‐5% (Figure 1d and Figure S6, Supporting Information). Furthermore, the energy‐dispersive X‐ray spectroscopy (EDS) element mapping images with the line profile show the homogeneously distribution of Zn in the nano‐octahedrons of precursor Cu_2_O–Zn‐1% and precursor Cu_2_O–Zn‐5% (Figure 1e,f). However, when the content of Zn is raised to 25%, numerous irregular nanoparticles are observed around the octahedral structured precursor Cu_2_O–Zn‐25% (Figure S7, Supporting Information). Further HRTEM and EDS element mapping images (Figure S8, Supporting Information) confirm that these irregular nanoparticles are zinc oxide while the octahedral particles are precursor Zn‐doped Cu_2_O, suggesting that only a small proportion of Zn can be doped into Cu_2_O and the excess Zn forms ZnO nanoparticles around precursor Zn‐doped Cu_2_O octahedrons.

**Figure 2:**
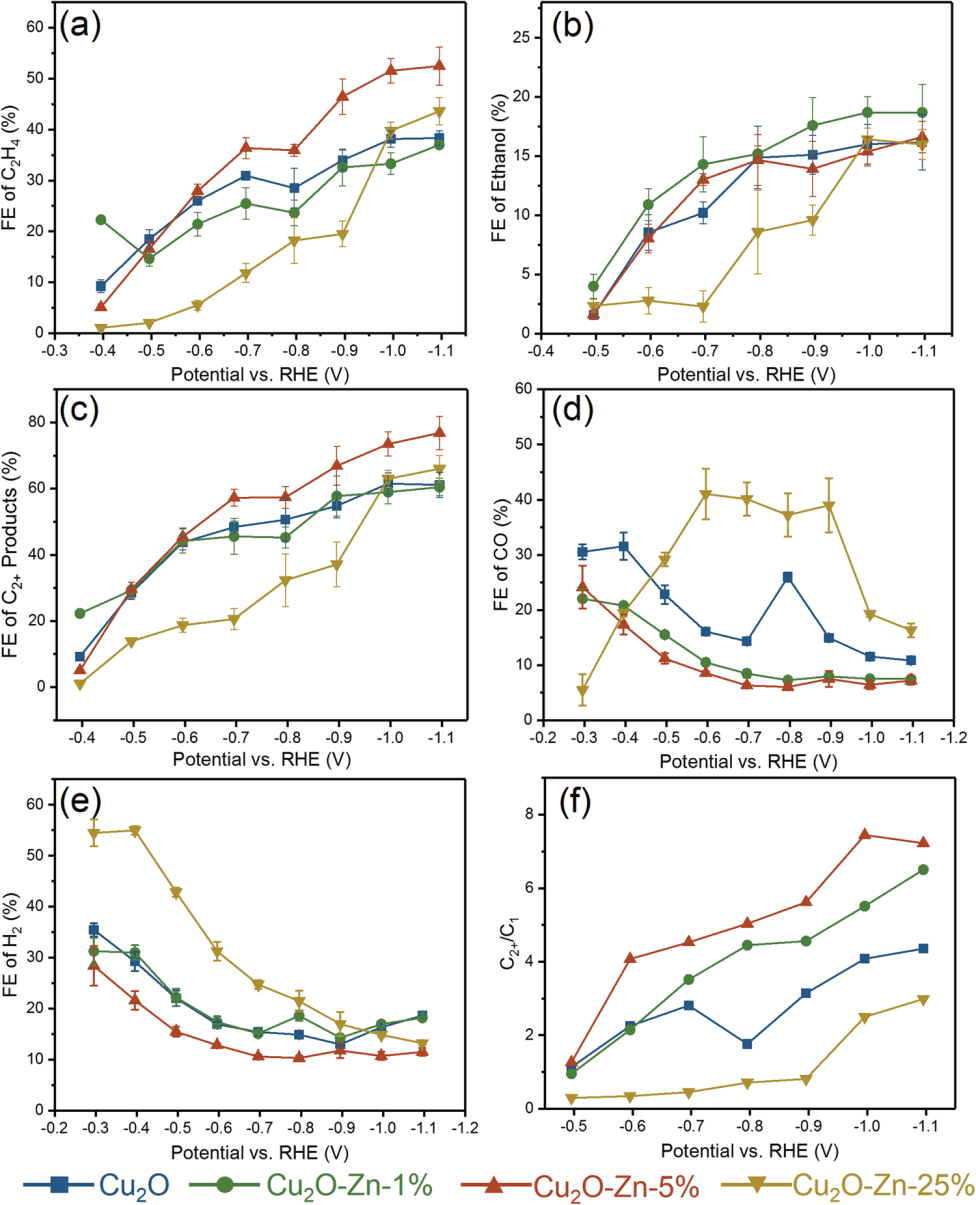
The Faradaic efficiency (FE) of a) C_2_H_4_, b) EtOH, c) C_2+_ products, d) CO, e) H_2_, and f) the ratio of C_2+_/C_1_ at different potentials over Cu_2_O, Cu_2_O–Zn‐1%, Cu_2_O–Zn‐5%, and Cu_2_O–Zn‐25% in 1 m KOH using a commercial gas diffusion electrode flow cell.

**Figure 3:**
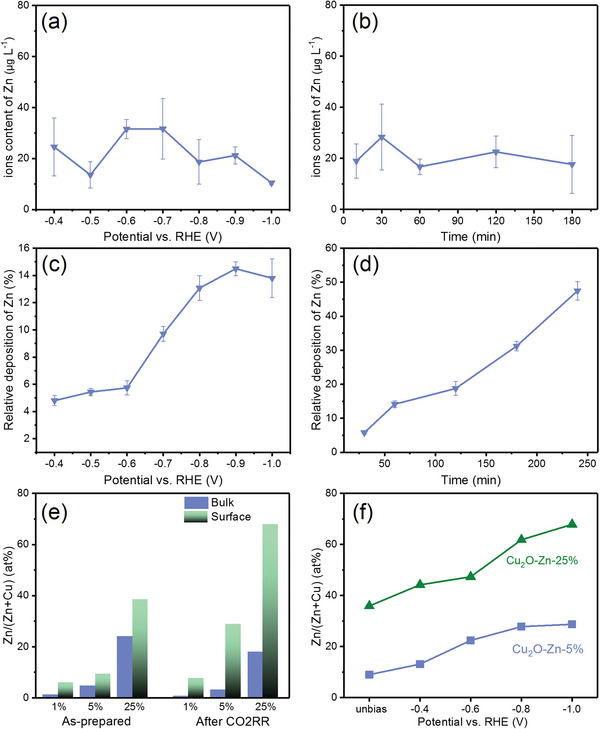
a) Potential‐dependent Zn^2+^ concentration using Cu_2_O–Zn‐5% as the catalyst after 30‐min CO2RR. b) Time‐dependent Zn^2+^ concentration using Cu_2_O–Zn‐5% as the catalyst at –0.9 V. c) Potential‐dependent relative deposition of Zn over Cu_2_O using 1 m KOH with 0.2 × 10^−3^ m Zn^2+^ as the catholyte after 60‐min CO2RR. d) Time‐dependent relative deposition of Zn over Cu_2_O using 1 m KOH with 0.2 × 10^−3^ m Zn^2+^ as the catholyte at –0.9 V. e) Bulk and surface atomic ratio of Zn and Cu on Cu_2_O–Zn catalysts before and after CO2RR. f) Surface atomic ratio of Zn and Cu on Cu_2_O–Zn catalysts under different potentials.

The CO2RR catalytic performance of the bare Cu_2_O and Zn‐doped Cu_2_O series catalysts in 1 m KOH solution is investigated using a commercial gas diffusion electrode‐based flow cell. The real active components are obtained by constant‐potential electrolysis under the bias of –0.7&nbsp;V for 0.5&nbsp;h. In Figure S9 (Supporting Information), the total current densities of the bare Cu_2_O and Cu_2_O–Zn series catalysts do not differ significantly and are independent of the Zn content. The maximum current densities (about **–**340 mA cm**^–^** ^2^) are obtained at **–**1.0 V, and the total current density does not increase when the potential is more negative than **–**1.0 V due to the mass transport limitations. Eight products from CO2RR are detected, including C_2_H_4_, EtOH, CO and H_2_ etc. (**Figure** **2** and Figure S10, Supporting Information). The maximum FEs of C_2_H_4_ are achieved at approximately −1.1 V, with values of 38%, 37%, 52%, and 43% for Cu_2_O, Cu_2_O–Zn‐1%, Cu_2_O–Zn‐5%, and Cu_2_O–Zn‐25%, respectively (Figure 2a). Notably, Cu_2_O–Zn‐5% exhibits the best selectivity for C_2_H_4_. In particular, Cu_2_O–Zn‐25% shows the smallest FE of C_2_H_4_ at the potentials positive than **–**1.0 V due to the higher coverage of Zn sites at the surface, which serve as active sites for competing CO generation.^[^ ^19^ ^]^ For ethanol, the maximum FEs of approximately 20% are achieved, with no significant difference observed among Cu_2_O–Zn series catalysts (Figure 2b). Other minor products, such as acetate, n‐PrOH, formate, and CH_4_, are also detected over Cu_2_O–Zn catalysts with FE less than 10% (Figure S10, Supporting Information). Moreover, the FEs of total C_2+_ products of all the samples increase with the applied potential negatively shifted. Specifically, a maximum total FE of 60% for C_2+_ products is achieved over the bare Cu_2_O catalysts at approximately **–**1.1 V (Figure 2c), which is consistent with the early reports,^[^ ^20^ ^]^ while the Cu_2_O–Zn‐5% exhibits a high FE of 77% for total C_2+_ products with the lowest FEs (**Figure** **3**d,e) of CO and H_2_, indicating that its improved FE of C_2+_ is mainly due to the inhibition of hydrogen evolution and CO generation. Consequently, as shown in Figure 2f, Cu_2_O–Zn‐5% promotes the C_2+_/C_1_ ratio of the products evidently. Moreover, the long‐term stabilities determined at **–**1.0 V exhibit a violent volatility (Figure S11, Supporting Information), resulting from the large number of O_2_ bubbles generated from the anode. Meanwhile, the FE of C_2_H_4_ displays slight decrease within 6 h due to the ineluctable flooding effect on the gas diffusion electrode. After a long‐term electrochemical test, the SEM images show that the structure of nano‐octahedral is roughly retained, and a large number of nanoparticles appear on the surface (Figure S12, Supporting Information), which can be explained by the degradation mechanisms.^[^ ^3^ ^]^ Moreover, TEM (Figure S13, Supporting Information) and XRD pattern (Figure S14, Supporting Information) also show the d‐spacing and the main peak of metallic Cu(111) plane after the electrolysis, respectively. Inductively coupled plasma‐optical mass spectrometer (ICP–MS) reveals the similar Cu/Zn ratios of Cu_2_O–Zn‐5% before and after the electrolysis (Figure S15, Supporting Information).

It is noted that Zn (ZnO) can be easily dissolved in strong alkaline electrolyte due to its nature as an amphoteric metal (oxide). In this regard, the present study conducted an investigation into the dissolution of Zn during the CO2RR by Cu_2_O–Zn‐5% electrodes. The Zn^2+^ concentration in the electrolyte (1 m KOH) was measured under different potentials after 30 min electrocatalysis by ICP–MS, as shown in Figure 3a. The result suggests that the Zn leaching from the electrode occurs in all the operation stages of CO2RR, with the Zn^2+^ ion concentration in the electrolyte showing a volcano‐type relationship with the applied negative potentials, reaching the peak concentration at –0.7 V. This trend is ascribed to the combined effects of the applied negative potentials and higher local pH at the surface of the electrodes, which are favorable for Zn redeposition and leaching, respectively. Besides, the time‐dependent Zn^2+^ concentration in electrolyte under the potential of –0.9 V (Figure 3b) also confirm the dissolution of Zn during CO2RR, with the periodic fluctuation of the Zn^2+^ content over the operation time, indicating the possible dynamic stability of the leaching and redeposition process. The atomic ratios of Zn in the bulk catalysts after CO2RR (Figure 3e) also show the lower content of Zn, which means the dynamic change of Zn‐doped Cu_2_O catalysts during CO2RR, i.e., Zn leaching into the electrolyte.

Moreover, to confirm the expected redeposition of Zn ions at the surface of catalysts in CO2RR, 1 m KOH with 0.2 × 10^−3^ m Zn^2+^ was used as the electrolyte for the bare Cu_2_O. The deposited Zn onto the electrode was detected by ICP–MS again, and the relative deposition of Zn was calculated by the reduction of Zn^2+^ concentration in the 1 m KOH with 0.2 × 10^−3^ m Zn^2+^ electrolyte. Figure 3c,d exhibits that the deposited Zn indeed happens and increases with the negative shift of the applied potentials and the operation time. The appearance of XPS peak of Zn 2p (Figure S16, Supporting Information) and the EDS element mapping images (Figure S17, Supporting Information) of the bare Cu_2_O after CO2RR confirm the depositional process of Zn^2+^ in CO2RR as well, supporting the dynamic surface reconstruction of Cu_2_O–Zn catalysts via the leaching and redeposition of Zn.

To further elucidate the composition change of Cu_2_O–Zn after such leaching and redeposition process in electrocatalysis, the bulk and surface atomic fractions of Cu and Zn before and after CO2RR were detected by ICP–MS and XPS, respectively. As shown in Figure 3f, the bulk atom ratios of Cu/Zn of the as‐prepared Cu_2_O–Zn series catalysts are almost consistent with the nominal fractions. However, the atomic fractions of Zn at the surface are much higher than those of the bulk of the Zn‐doped Cu_2_O catalysts, indicating that the Zn could be preferentially segregated to the surface, which can be attributed to the surface energy minimization principle by the lower surface energy of Zn.^[^ ^21^ ^]^ After CO2RR, the atomic fraction of Zn in the bulk decreases slightly, which may originate from the leaching of Zn or ZnO in strong alkaline electrolyte and higher local pH (Figure 3c). However, the surface atomic Zn increases significantly after CO2RR, indicating dynamic reconstruction by the leaching (from both bulk and surface) and redeposition of Zn onto the surface of Cu_2_O. In addition, the surface atomic fraction of Zn also increases with the negative shift of potential on Cu_2_O–Zn‐5% and Cu_2_O–Zn‐25% (Figure 3f), consistent with the above conclusion of potential‐dependent deposition of Zn over Cu_2_O (Figure 3d).

**Figure 4:**
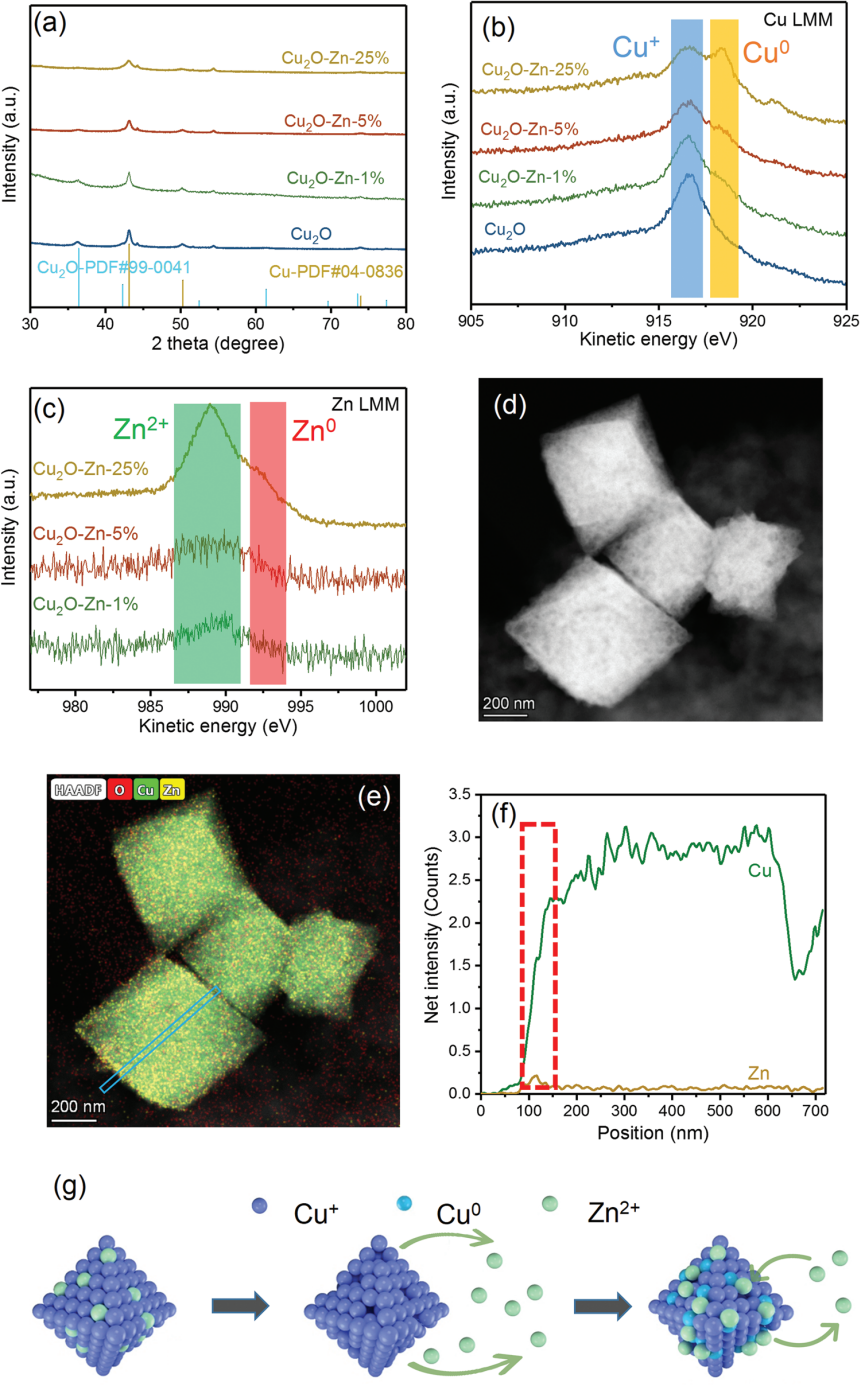
a) X‐ray diffraction (XRD) patterns and Auger electron spectroscopy (AES) spectra of b) Cu LMM and c) Zn LMM of Cu_2_O, Cu_2_O–Zn‐1%, Cu_2_O–Zn‐5%, and Cu_2_O–Zn‐25% after 1‐h CO2RR at –1.0 V. d) High angle angular dark field‐scanning transmission electron microscopy (HAADF–STEM) with corresponding e) energy‐dispersive X‐ray spectroscopy (EDS) element mapping images and f) line profile of Cu_2_O–Zn‐5% after 1‐h CO2RR at –1.0 V. g) Scheme for dissolution and redeposition mechanism of Cu_2_O–Zn catalysts under CO2RR.

The composition change could lead to the tuning of the structure and chemical state, which are crucial for enhancing CO2RR performance. The XRD patterns of the catalysts were obtained immediately after CO2RR. As shown in **Figure** **4**a, all the samples exhibit strong diffraction peaks of metallic Cu and weak peaks of Cu_2_O, suggesting that Cu_2_O component is essentially reduced to Cu^0^ in bulk during CO2RR. Nevertheless, no phases of metallic Zn or ZnO can be observed in Cu_2_O–Zn series catalysts after CO2RR (even in Cu_2_O–Zn‐25%), indicating that a significant portion of Zn have either leaching in the strong alkaline electrolyte or highly dispersed. Notably, the surface chemical states of Cu_2_O–Zn series catalysts differ significantly from those in the bulk after CO2RR. As shown in Figure 4b, in the LMM results, there is almost no obvious characteristic peaks of Cu^0^ on the bare Cu_2_O and Cu_2_O–Zn‐1% catalysts after the electrocatalysis, instead, the peaks of Cu^+^ are dominant at the surface of these two samples, while a small Cu^0^ peak is present on Cu_2_O–Zn‐5%, and Cu_2_O–Zn‐25% displays the highest intensity of Cu^0^ peak. These data suggest that Zn doping can increase the ratio of Cu^0^ at the reconstructed surface via the dynamic stability. Moreover, the potential‐dependent Cu LMM spectra (Figure S18, Supporting Information) also confirm that the Cu^0^ peaks after CO2RR evidently appear in the Zn‐doped Cu_2_O samples but not in the bare Cu_2_O. These results show that Zn doping leads to more Cu^0^/Cu^+^ interface that is beneficial for the efficient CO_2_ conversion to C_2+_ products, since Cu^0^ activates CO_2_ and facilitates the following electron transfers, and Cu^+^ strengthens *CO adsorption to further boost C−C coupling.^[^ ^22^ ^]^ The previous reports described that ZnO can be thoroughly reduced to metallic Zn under CO2RR condition,^[^ ^23^ ^]^ however, in our case, most Zn still shows the chemical state of Zn^2+^, attributed to the strong oxidation capacity of the alkaline electrolyte and high local pH.^[^ ^8^ ^]^ Only small peaks of Zn^0^ in Zn 2p (Figure S19, Supporting Information) and Zn LMM Auger spectra (Figure 4c) can be observed. According to the standard electrode potentials of Zn^2+^/Zn and Cu^+^/Cu:^[^ ^24^ ^]^

(1) $${\mathrm{Zn}}^{2+}+2e^{-}↔\mathrm{Zn}\left(s\right)E^{0}=-0.76\;V\;\mathrm{vs}.\;\mathrm{SHE}$$

(2) $${\mathrm{Cu}}^{+}+e^{-}↔\mathrm{Cu}\left(s\right)\;\;\;E^{0}=0.52\;V\;\mathrm{vs}.\;\mathrm{SHE}$$

Therefore,

(3) $${\mathrm{Cu}}^{+}+\mathrm{Zn}\left(s\right)↔\mathrm{Cu}\left(s\right)+{\mathrm{Zn}}^{2+}+e^{-\;}E^{0}=1.28\;\;V\;\mathrm{vs}.\;\mathrm{SHE}$$

In our case, once the Zn^2+^ is reduced to metallic Zn^0^, it is prone to reduce Cu_2_O to Cu^0^ subsequently, thus the proportion of Cu^0^ can be increased within the content of Zn of the Cu_2_O–Zn catalysts. Therefore, the leaching and redeposition mechanism can provide rich Cu–Zn sites at the surface, which plays an important role in improving the selectivity of C_2+_ products (Figure 4g).

Meanwhile, the morphology of Zn‐doped Cu_2_O nano‐octahedrons has been changed after CO2RR, which is confirmed by SEM and HRTEM images. Figure S20a–d (Supporting Information) shows the SEM images of the bare Cu_2_O and Zn‐doped Cu_2_O catalysts after 1‐h CO2RR under a potential of –1.0 V, which depicts that the octahedron structures of the bare Cu_2_O, Cu_2_O–Zn‐1%, and Cu_2_O–Zn‐5% catalysts are largely retained during the electrolysis. However, some nanosheets emerge at the surface of the bare Cu_2_O, while the octahedral structures of Cu_2_O–Zn‐1% and Cu_2_O–Zn‐5% transform into aggregated nanoparticles with sizes of several dozen nanometers. HRTEM images of Cu_2_O–Zn series catalysts after CO2RR (Figure S16e–h, Supporting Information) show abundant lattice boundaries, which are found to facilitate the adsorption of the key intermediate (*CO) at the catalyst surface, thereby boost the further CO2RR into C_2+_ products.^[^ ^25^ ^]^ Moreover, the EDS element mapping images of Cu_2_O–Zn‐1% (Figure S21, Supporting Information) confirm that Cu, Zn, and O remain evenly distributed in the octahedrons after CO2RR. In contrast, Cu_2_O–Zn‐5% after CO2RR possesses the higher Zn content at the surface of the nano‐octahedron than in the interior (Figure 4d,e, and the red marking area in Figure 4f), which is consistent with the results obtained from the investigation on the surface atom ratio (Figure 3f).

**Figure 5:**
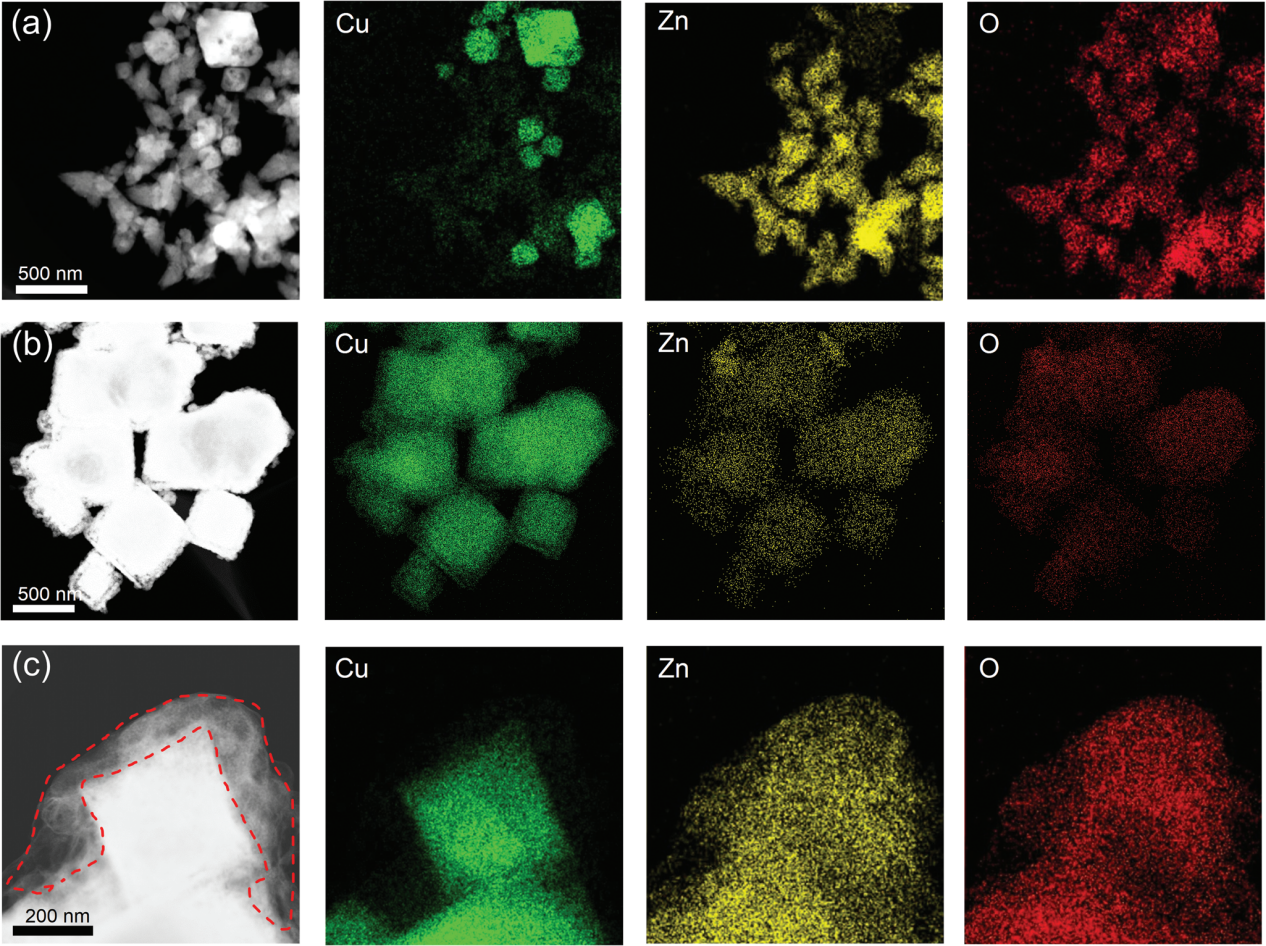
Scanning transmission electron microscopy (STEM) image and corresponding energy‐dispersive X‐ray spectroscopy (EDS) element mapping images of Cu_2_O–Zn‐25% a) before CO2RR, after 1‐h CO2RR under b) –0.7 V and c) –1.0 V.

Intriguingly, from the scanning transmission electron microscopy (STEM) and EDS‐mapping images (**Figure** **5**b,c), the thickness of the ZnO layer coating on Cu_2_O nano‐octahedrons in Cu_2_O–Zn‐25% after CO2RR increases as the applied potential shifts from –0.7 to –0.9 V. It is noted that the disappearance of a large number of separate ZnO nanoparticles after CO2RR (Figure 5a) provided further evidence of the dynamic stability process that ZnO nanoparticles in the material are gradually dissolved and redeposited at the surface. This also explains the main products of CO and H_2_ by Cu_2_O–Zn‐25% at the more positive potentials, and the sharply increased FE of C_2_H_4_ with the negative shift of the potentials (Figure 2a,c). At more positive potentials, a large number of ZnO nanoparticles participate in the reaction as the active sites of electrocatalytic CO2RR to CO. However, with the negative shift of the potential, ZnO is gradually leaching and redeposited on the surface of Cu_2_O, creating rich Cu–Zn bimetallic sites. These bimetallic sites have been proven to influence the interaction between the key intermediate *CO and the Cu–Zn sites, which affects the electron transfer between the *CO and active sites and enhance the selectivity of electrocatalytic CO2RR for the C_2+_ products,^[^ ^26^ ^]^ so that the FE of C_2+_ products better than the bare Cu_2_O is obtained under **–**1.0 and **–**1.1 V. To further investigate the effects of Cu–Zn bimetallic sites caused by leaching and redeposition, the physical mixing of Cu_2_O and ZnO with 5 at% Zn (named physical‐mixed Cu_2_O–Zn‐5%) is prepared. As shown in Figure S22a (Supporting Information), the physical‐mixed Cu_2_O–Zn‐5% shows the higher FE of CO (above 20%) compared with the Cu_2_O catalyst due to the isolated Zn sites (without Cu–Zn binding), and the max C_2+_ products selectivity is only 54.2% at **–**1.1 V. Here, it is noted that the products after a short time of 10‐min electrolysis are collected to avoid the impact of reconstruction. However, after potentiostatic activation at **–**1.0 V for 1 h, the leaching and redeposition progress of Zn induces the excellent CO2RR performance with a C_2+_ products FE of 65.5%, much higher than that of the fresh physical‐mixed Cu_2_O–Zn‐5% and bare Cu_2_O catalysts (Figure S22b, Supporting Information). Thus, the isolated ZnO is dissolved and redeposited on the surface of Cu_2_O to build the Cu–Zn bimetallic sites, which play an essential role in increasing the selectivity of C_2+_ products.

Besides, the structural evolution of the Cu_2_O–Zn‐5% catalyst during CO2RR is investigated under different potentials by quasi‐operando XRD (Figure S23, Supporting Information). Under negative potentials, the diffraction peaks of Cu *fcc* phase become more prominent, whereas the peak intensity of Cu_2_O gradually decreases with the negative shift of the potentials. Moreover, Figure S24 (Supporting Information) depicts the morphological changes in Cu_2_O–Zn‐5% nano‐octahedrons after 1 h of CO2RR at various potentials. As potential goes negatively, the surface of the octahedrons becomes coarser and more porous. Ultimately, the surface of Cu_2_O–Zn‐5% nano‐octahedrons could be partially decomposed into small nanoparticles under high negative applied potentials, as shown in the SEM images of the sample under **–**1.0 V. This further dissociation from large octahedrons to small nanoparticles can provide abundant surface undercoordinated surface sites, which are known to be active sites for CO_2_ activation and further catalytic conversion.^[^ ^27^ ^]^

Furthermore, in order to generalize the findings of the dynamic stability of surface reconstruction to other amphoteric metal‐doped catalysts, 5 at% Al‐doped Cu_2_O was prepared and investigated for CO2RR as the electrocatalyst. The multiple correlative characterizations, including XRD (Figure S25, Supporting Information), XPS, Cu LMM spectra (Figure S26, Supporting Information), and high angle angular dark field STEM (HAADF–STEM) with corresponding EDS mapping images (Figure S27, Supporting Information), confirm the successful doping of Al into Cu_2_O. Similar to Zn, the leaching and redeposition of Al has been also confirmed by ICP–MS results during CO2RR (Figure S28, Supporting Information), and the Al^3+^ content in electrolyte is one order of magnitude higher than Zn^2+^, indicating a faster leaching rate of Al. Moreover, the highest Al^3+^ content is detected at the operation time of 30 min, and then it decreases, suggesting that dominant leaching progress at the initial stage of CO2RR is replaced by the subsequent dominant deposition process of Al^3+^. A maximum FE of 66% for C_2+_ products is achieved over Al‐doped Cu_2_O catalyst at **–**1.1 V (Figure S29, Supporting Information), which is higher than that of Cu_2_O alone (60%). These results reveal the potential for amphoteric metal (oxide) catalysts to provide rich surface bimetallic sites through the dynamic stability of leaching and redeposition, indicating that the electrochemical surface reconstruction of these catalysts cannot be ignored for electrocatalytic reactions.

**Figure 6:**
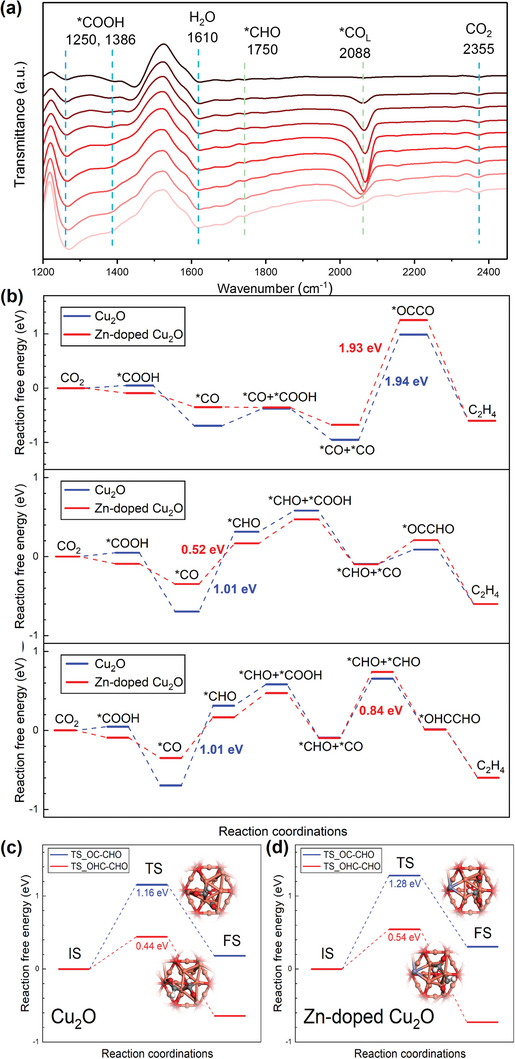
a) In situ attenuated total reflection Fourier‐transform infrared spectroscopy (ATR–FTIR) spectra on Cu_2_O–Zn‐5% at a potential range from −0.4 to −1.4 V. b) From top to bottom: Free‐energy diagrams for CO2RR to C_2_H_4_ on the Cu_2_O(111) surface and Zn‐doped Cu_2_O(111) surface via OC–CO pathway, OC–CHO pathway, and OHC–CHO pathway, respectively. Activation barrier diagram of C–C coupling via OC–CHO and OHC–CHO pathways on c) Cu_2_O and d) Zn‐doped Cu_2_O. The structures of the transition states are presented. IS: initial state, TS: transition state, FS: final state. Gray, white, violets, orange, and red balls represent C, H, Zn, Cu, and O, respectively.

To gain further insight into the reaction mechanism on Cu_2_O–Zn catalyst, in situ ATR–FTIR was conducted to detect the adsorbed intermediates on Cu_2_O–Zn‐5% during the CO2RR. As shown in **Figure** **6**a, the peak around 2355 cm^−1^ corresponds to the stretching vibration of CO_2_, and the peak around 1617 cm^−1^ belongs to the deformation band of H_2_O.^[^ ^28^ ^]^ With the negative shift of potential, the peaks appeared at 1250 and 1390 cm^−1^ can be attributed to the OH deformation and C–O stretch of *COOH, respectively, which is generally considered a crucial intermediate in the production of various products. The peak at 2088 cm^−1^ that emerges with the negative shift of potential is attributed to the C≡O stretching mode of linearly bonded CO species (*CO_L_), showing a trend of first increasing and then decreasing.^[^ ^29^ ^]^ This result indicates the initial formation of CO intermediates and subsequent transmutation into other hydrocarbons at more negative bias. Moreover, the increased peak of stretching of adsorbed *CHO intermediate (1750 cm^−1^)^[^ ^30^ ^]^ suggests that the possible C–C coupling for formation of C_2_H_4_ could occur via the OC–CHO pathway or OHC–CHO pathway on Cu_2_O–Zn‐5% catalyst.

The calculated charge density difference of the Zn‐doped Cu_2_O in Figure S30 (Supporting Information) obviously shows that electrons are depleted around dopant Zn and accumulated in the Cu_2_O surface, indicating the charge transferred from Zn to Cu. This can result in the Cu^+^ reduced to a lower valence state and causing an easier reduction reaction on the Cu site, which agrees well with the XPS result after CO2RR (Figure 4b). In order to determine the thermodynamic and kinetic feasibility of the mechanism, DFT calculations were further conducted. The results indicate three possible reaction mechanisms based on different C–C coupling processes, namely OC–CO pathway, OC–CHO pathway, and OHC–CHO pathway (Figure 6b and Table S1, Supporting Information). The first step in all of these pathways involves the hydrogenation of the gas‐phase CO_2_ molecule to form a *COOH intermediate, with the C atom locating at an unsaturated Cu site. This intermediate is then reduced to H_2_O and a *CO intermediate, which remains at the Cu site. In the case of the OC–CO pathway, a second CO_2_ molecule is subsequently reduced to *CO at another unsaturated Cu site (Figures S31 and S32, Supporting Information), leading to the formation of two *CO intermediates. The C–C coupling then occurs between these two *CO to produce C_2_H_4_. However, the reaction free energies of this C–C coupling process on both Cu_2_O and Zn‐doped Cu_2_O are found to be 1.94 and 1.93 eV, respectively. The difference in energy between the two catalysts is negligible, suggesting that the presence of Zn does not significantly affect the reaction. Moreover, the relatively high reaction free energy of approximately 2 eV makes this process challenging. As such, the formation of C_2_H_4_ via the OC–CO pathway could not be feasible. In the case of the OC–CHO and OHC–CHO pathways, the *CO intermediate undergoes further reduction to form the *CHO intermediate. It is observed that the *CO adsorption energy on Cu_2_O is significantly lower than that on Zn‐doped Cu_2_O, resulting in a higher energy requirement for the hydrogenation of *CO on Cu_2_O to overcome its stable adsorption. This leads to the formation of *CHO with a reaction free energy of 1.01 eV on Cu_2_O, which is higher than 0.52 eV observed on Zn‐doped Cu_2_O. The subsequent C–C coupling reactions between the *CO and *CHO intermediates on both Cu_2_O and Zn‐doped Cu_2_O exhibit free energy uphills of 0.18 and 0.31 eV, respectively, which are lower than that required for the hydrogenation of *CO. Therefore, it is determined that RDS of the OC–CHO pathway on both Cu_2_O and Zn‐doped Cu_2_O are the hydrogenation of *CO to *CHO. This sluggish step leads to the accumulation of *CO, which is also confirmed by the in situ ATR–FTIR results (Figure 6a). In addition to the OC–CHO pathway, the C–C coupling reaction can also occur between two *CHO intermediates through the OHC–CHO pathway. In this pathway, the adsorbate *CO can be further reduced to another *CHO intermediate. The reaction free energies of the second *CHO formation on Cu_2_O and Zn‐doped Cu_2_O are found to be 0.75 and 0.84 eV, respectively. The subsequent C–C coupling reaction to form *OHCCHO is exothermic, enabling it to occur spontaneously. Thus, in the OHC–CHO pathway, the RDS on Cu_2_O remains the first hydrogenation of *CO with a reaction free energy of 1.01 eV, while on Zn‐doped Cu_2_O it is the second hydrogenation of *CO with a reaction free energy of 0.84 eV. Following the formation of *OCCHO and *OHCCHO, the subsequent reduction reaction leading to the formation of C_2_H_4_ happens spontaneously. In both the OC–CHO pathway and the OHC–CHO pathway, the reaction free energies of the RDS on Zn‐doped Cu_2_O are lower than that on Cu_2_O, suggesting that the presence of Zn dopant in Cu_2_O results in improved performance of CO_2_ reduction to C_2_H_4_ formation. In order to determine the actual reaction pathway, the transition states and activation barriers for these two C–C coupling processes were systematically investigated. As shown in Figure 6c,d, the activation barrier for the *OC–CHO process on Cu_2_O is found to be 1.16 eV, which is significantly higher than that for the *OHC–CHO process (0.44 eV). Similar results are obtained for the Zn‐doped Cu_2_O catalyst. On the basis of these kinetic energy barriers, it is concluded that the reduction of CO_2_ to C_2_H_4_ primarily occurs via the OHC–CHO pathway.

## 3 Conclusion

In this study, Zn‐doped Cu_2_O nano‐octahedrons are prepared and used for investigating the leaching and redeposition process of the amphoteric metal for CO2RR. Cu_2_O–Zn‐5% shows excellent activity and selectivity for CO2RR toward C_2+_ products (FE of 77% with a current density of –340 mA cm^–2^ at −1.0 V in a commercial flow cell), while the productions of CO and H_2_ are significantly inhibited. Moreover, the leaching and redeposition mechanism of Zn, which is influenced by the strong alkaline electrolyte and the reduction potentials, has been confirmed. The reconstructed surface of Zn‐doped Cu_2_O catalysts provided abundant Cu–Zn sites, as evidenced by the increased surface atomic ratio of Zn. Meanwhile, the ratio of metallic Cu^0^ in Zn‐doped Cu_2_O catalysts increases by the increased reconstructed‐surface Zn, leading to more Cu^0^/Cu^+^ interfaces and improved selectivity of C_2+_ products. Similar observations can be made with Al‐doped Cu_2_O from CO2RR as well. In situ ATR–FTIR and DFT calculations reveal that the C–C coupling processes on bare Cu_2_O and Cu_2_O–Zn primarily occur through the OHC–CHO pathway, and the introduction of Zn into Cu could efficiently lower the energy barrier for CO2RR to C_2_H_4._ This study not only presents an effective strategy for designing efficient Cu‐based catalysts for CO2RR to C_2+_ products, but also sheds new light on the surface reconstruction of amphoteric metal‐doped Cu_2_O catalysts.

## Conflict of Interest

The authors declare no conflict of interest.

## Supporting information

Supporting Information

## Data Availability

The data that support the findings of this study are available in the supplementary material of this article.
