# Supplementary material for: Dynamic Surface Reconstruction of Amphoteric Metal (Zn, Al) Doped Cu2O for Efficient Electrochemical CO2 Reduction to C2+ Products
Source: Adv Sci (Weinh). 2023 Aug 2;10(28):2303726. doi: 10.1002/advs.202303726 (PMC10558649; doi:10.1002/advs.202303726)
Supplement: Supplementary file 1 — Supporting Information [file ADVS-10-2303726-s001.pdf]

ADVANCED SCIENCE
Open Access

Supporting Information

for *Adv. Sci.*, DOI 10.1002/advs.202303726

Dynamic Surface Reconstruction of Amphoteric Metal (Zn, Al) Doped Cu_2O for Efficient Electrochemical CO_2 Reduction to C_{2+} Products

Yufei Jia, Yunxuan Ding, Tao Song, Yunlong Xu, Yaqing Li, Lele Duan, Fei Li, Licheng Sun and Ke Fan**

Supporting Information

Dynamic Surface Reconstruction of Amphoteric Metal (Zn, Al)-Doped Cu_2O for Efficient Electrochemical CO_2 Reduction to C_{2+} Products

Yufei Jia#, Yunxuan Ding#, Tao Song, Yunlong Xu, Yaqing Li, Lele Duan, Fei Li, Licheng Sun*, Ke Fan*

[a] Yufei Jia, Yulong Xu, Yaqing Li, Fei Li, Licheng Sun and Ke Fan
State Key Laboratory of Fine Chemicals, Institute of Artificial Photosynthesis, DUT-KTH Joint Education and Research Centre on Molecular Devices, Institute for Energy Science and Technology, Dalian University of Technology, 116024, Dalian, China
E-mail: *sunlicheng@westlake.edu.cn; kefan@kth.se*, *kefan@dlut.edu.cn*

[b] Yunxuan Ding, Licheng Sun
Center of Artificial Photosynthesis for Solar Fuels and Department of Chemistry, School of Science, Westlake University, 310024 Hangzhou, P. R. China

[b] Tao Song, Lele Duan
Department of Chemistry and Shenzhen Grubbs Institute, Southern University of Science and Technology, Shenzhen 518055, P.R. China

# These authors contribute equally to this paper.

Methods

Material characterizations

Rigaku diffractometer (Japan) equipped with monochromatized Cu Kα radiation (λ = 0.15418 nm) was used to conduct XRD measurement. Renishaw microscopic confocal Raman spectrometer (in Via Qontor) with a laser wavelength of 532 nm was used to conduct ex situ Raman measurement. XPS was performed through a Thermo XPS spectrometer system (ESCALAB XI+) using Al K_{α} (1486.6 eV, 150 W) radiation to research the materials compositions and valence states. Surface morphology was observed on a scanning electron microscopy (SEM, JEOL JSM-SU8220). Transmission electron microscopy (TEM) and high-resolution transmission electron microscopy (HRTEM) analysis were conducted by a JEM-2100F transmission electron microscope (JEOL, Japan) at an accelerating voltage of 200 kV. The amount of Cu and Zn in the catalyst were determined by inductively coupled plasma-optical mass spectrometer (ICP-MS). The gas products (H_2, CO, CH_4 and C_2H_4) were detected by gas chromatography (SHIMADZU, GC-2014). The liquid products (formate, EtOH, acetate and n-PrOH) were detected by ^{1}H nuclear magnetic resonance (NMR) spectroscopy spectrometer (500 MHz, Bruker Avance), with water suppression using a presaturation method.

Preparation of Cu_2O nano-octahedrons

In a typical procedure, 1 mmol $CuCl_2·2H_2O$ and 200 mg polyethylene glycol 20 000 were added to 200 mL deionized water and stirring for 5 min. Then, 1.2 mL 6 M NaOH was added into the above solution slowly. A blue precipitate of $Cu(OH)_2$ was

soon produced. After stirring for another 15 min, it was then added drop-wise with 1.5 mL hydrazine hydrate in 10 mL deionized water. The $Cu(OH)_2$ blue precipitates gradually turned into a brick red color and then black after stirring for 1 h. The final products were washed three times by the mixture of water and ethanol (volume ratio 1: 1) and dried in a vacuum oven at 50 °C for 6 h to obtain Cu_2O nano-octahedrons.

Preparation of Zn-doped Cu_2O nano-octahedrons

Zn-doped Cu_2O nano-octahedrons were prepared by the same preparation procedure of Cu_2O nano-octahedrons, except that the total amount of Cu precursor ($CuCl_2 \cdot 2H_2O$) and Zn precursor ($Zn(NO)_3 \cdot 6H_2O$) was maintained at 1 mmol with varied molar ratios of Cu: Zn (100: 0, 99: 1, 95: 5, and 75: 25, denoted as Cu_2O, Cu_2O-Zn-1%, Cu_2O-Zn-5%, and Cu_2O-Zn-25%, respectively).

Preparation of Al-doped Cu_2O nano-octahedrons

The obtain Cu_2O nano-octahedrons were dispersed in 5 mL of ethanol. $AlCl_3$ with an atomic ratio of 5% was dissolved in 5 mL of water, then added to the Cu_2O dispersion, and stirred for 3 h. The final products were washed three times with distilled water and ethanol, and dried in a vacuum oven at 50 °C for 6 h.

Preparation of ZnO nanoparticles

ZnO nanoparticles were prepared by the same preparation procedure of Zn-doped Cu_2O nano-octahedrons but without the Cu precursor.

Electrochemical measurements

The electrochemical performance was investigated using a CHI 760E electrochemical workstation (Chenhua, Shanghai) in a custom-designed gas diffusion

electrode-based flow cell with an anion exchange membrane as the separator, where the active catalysts loaded on carbon paper, platinum foil and saturated KCl (Ag/AgCl) were used as the working electrode, counter electrode and reference electrode, respectively. 1 M aqueous KOH served as the electrolyte for CO2RR activity test. All applied potentials were converted with respect to reversible hydrogen electrode (RHE).

$$E_{RHE} = E_{Ag/AgCl} + 0.059pH + E_{Ag/AgCl}$$

The working electrode was prepared as follows: 1 mg catalyst and 6 μL 5 wt% Nafion115 solution were dispersed in 80 μL ethanol and 160 μL water with sonication for 0.5 h to form a homogeneous ink. The ink was then dropcast onto a 3 × 2 cm^2 gas diffusion electrode (YLS-30T) with 2.0 × 0.5 cm^2 real work area, and then dried at room temperature before electrochemical tests.

The FE of gas and liquid products were calculated according to the following equation:

$$FE\ (\%) = \frac{Q_{products}}{Q_{tot}} = \frac{NFn}{It}$$

where n is the number of moles of gas and liquid products which can be calculated according to gas chromatography and NMR data, respectively; N represents the electron numbers transferred for one molecule of the product; F is the Faradaic constant (96485 C mol^{-1}); and Q_{tot} is the total charge passed during the electrolysis process; I is the current density; t is the electrolysis time.

In situ electrochemical ATR-FTIR measurements.

In situ electrochemical ATR-FTIR measurements were carried out by utilizing a Bruker Vertex 80 infrared spectrometer equipped with a liquid nitrogen-cooled RT-DLaTGS

detector. A hemisphere silicon prism was first polished with diamond abrasion paste and sonicated with deionized water. The prim then was immersed in a piranha solution (V_{H2SO4}/V_{H2O2} = 3/1) for 1 h. After this, three layers of metal are deposited on the pretreated silicon prism from bottom to top, which was conducted by an electron beam evaporation system (HHV, TF500), wherein the first layer is Cr (1.5 nm) and followed by Au (45 nm) and Ti (3 nm). The Cu_2O-Zn-5% ink slurry was spincoated onto the above metal-deposited silicon prism using a spin coater, and then was assembled into a three-electrode spectroelectrochemical cell as the working electrode. A Pt foil and an Ag/AgCl (saturated with KCl) were served as counter electrodes and reference electrodes, respectively. A constant CO_2 flow was continuously purged into the 0.1 M KHCO3 electrolyte during the experiment. In a typical test, the working electrode was reduced to a stable state by i-t measurements at –1.0 V within 1h. The in situ ATR-FTIR spectra were collected by sweeping the applied potential from –0.4 V to –1.4 V at a scan rate of 2.5 mV s^{-1} with a resolution of 15 s per spectrum at a spectral resolution of 4 cm^{-1} .

Computational details

All the DFT calculations in the work were carried out using the Vienna *ab initio* simulation program (VASP)[1]. The projector-augmented wave (PAW) method[1b, 2] was utilized to describe the core-valence interactions, and the plane-wave basis expansion cut-off energy was set to 500 eV. For exchange and to correlate the functional, the generalized gradient approximation (GGA) was used with Perdew–Burke–Ernzerhof (PBE)[3] to perform all spin-polarized calculations. The van der Waal (vdW) interaction

was described by the DFT-D3 method[4]. The equilibrium was reached when the forces on the relaxed atoms and the energies in the self-consistent iterations became less than 0.03 eV/Å and 10^{-5} eV, respectively. The theoretical lattice constant is calculated to be 4.26 Å, which agrees well with the experimental data of 4.27 Å[5]. For the model construction, a $p(2 \times 2)$ $Cu_2O(111)$ surface with four layers was modeled, in which the bottom two layers were kept fixed to mimic the bulk region while the other layers were allowed to fully relax. A ~15 Å vacuum layer was used to eliminate the interaction between neighboring slabs. The Zn-doped Cu_2O was formed by replacing one Cu atom in the top surface with one Zn atom. The model employed in this study was partially reduced and constructed with Cu and Cu_2O. A 5 × 5 × 5 and a 2 × 2 × 1 Monkhorst–Pack k-point mesh sampling were used for the bulk optimization and the surface optimizations, respectively. The transition states (TSs) were located with a constrained minimization technique[6] with the forces on the relaxed atoms less than 0.05 eV/Å.

The Gibbs free energy can be expressed as:

$$\Delta G = \Delta E + \Delta ZPE - T \cdot \Delta S \qquad (4)$$

where ΔE is the reaction energy calculated by the DFT methods. ΔZPE and $T\Delta S$ are the thermodynamic corrections of zero-point-energy (ZPE) and entropy (S) derived from the vibrational partition function at 298.15 K, respectively.

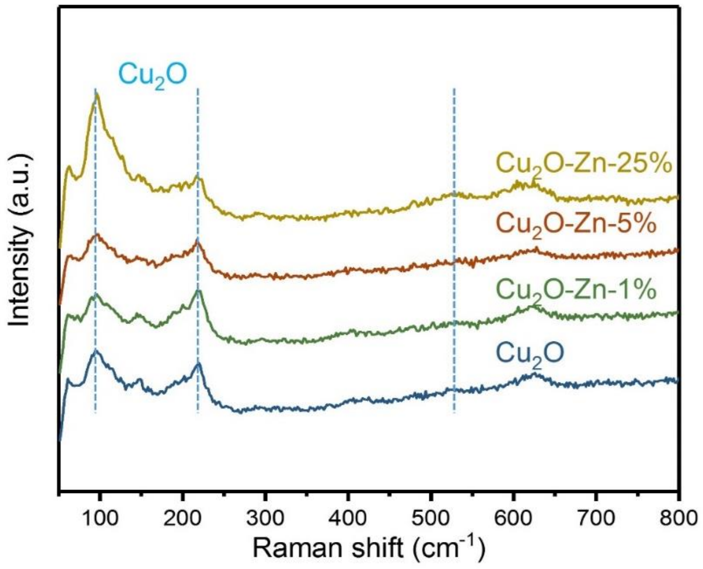

Figure S1. Raman spectra of the Zn-doped Cu_2O samples.

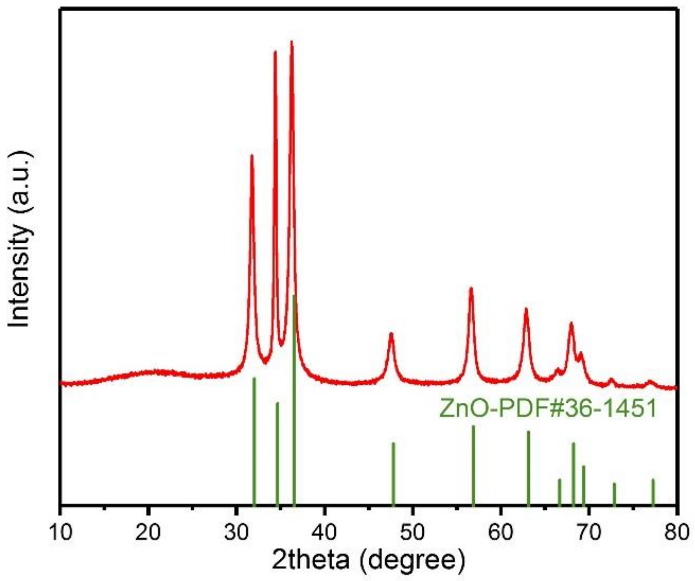

Figure S2. XRD pattern of ZnO nanoparticles.

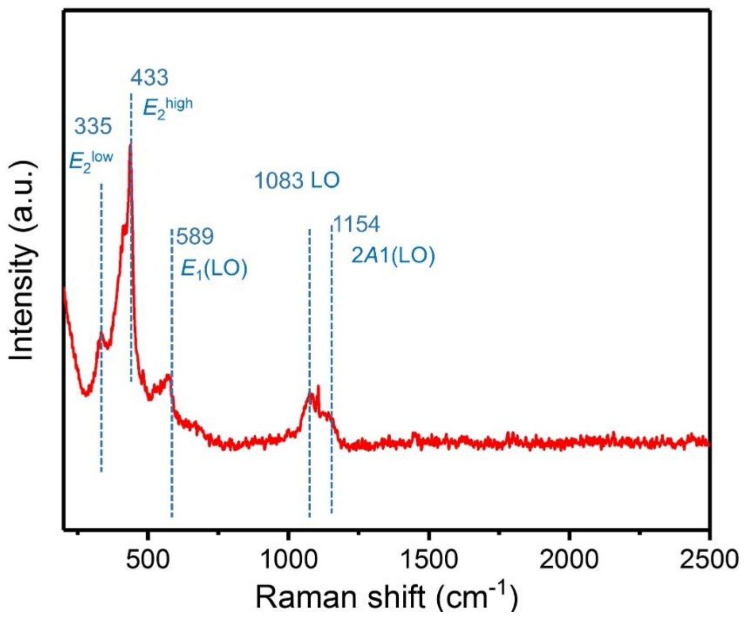

Figure S3. Raman spectrum of the bare ZnO sample.

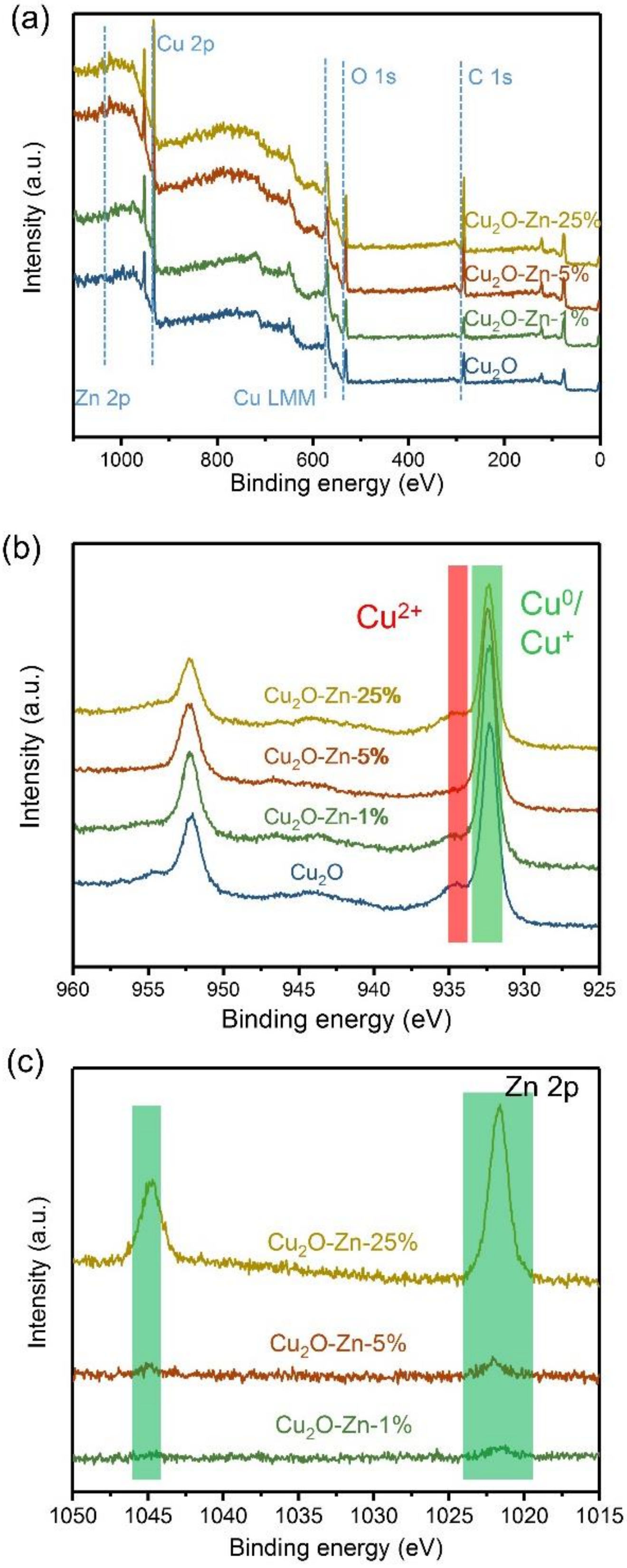

Figure S4. (a) XPS survey spectra, high-resolution XPS of (b) Cu 2p and (c) Zn 2p of the as-prepared Cu_2O, Cu_2O-Zn-1%, Cu_2O-Zn-5% and Cu_2O-Zn-25%.

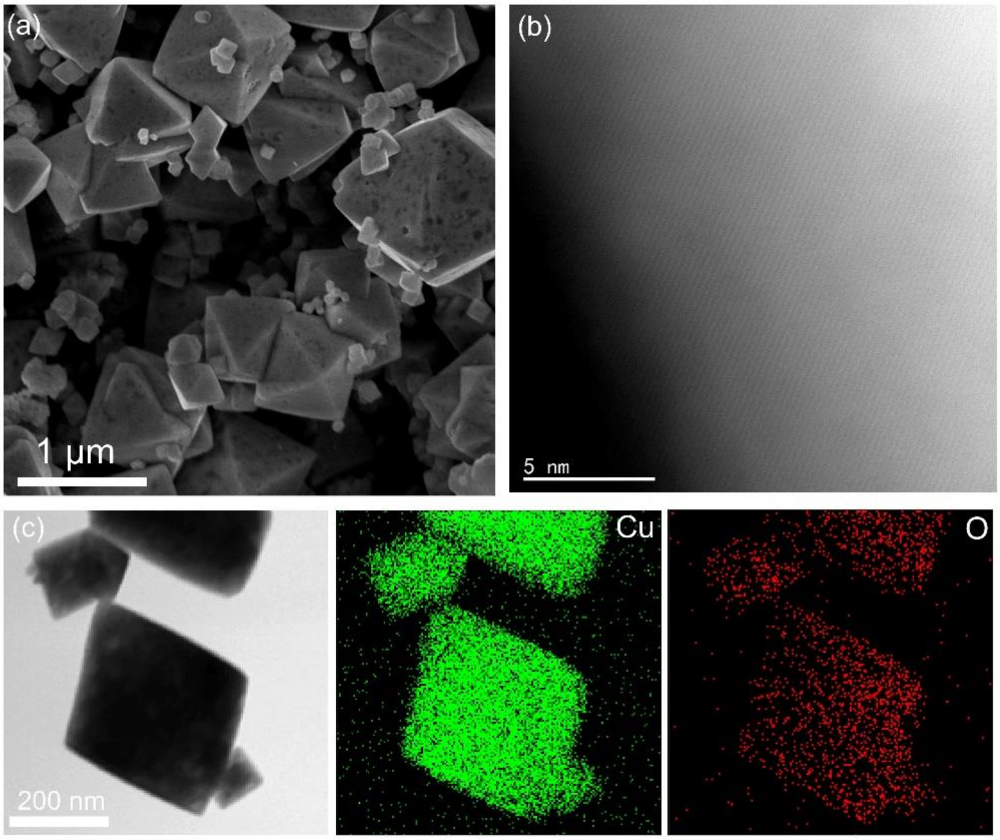

Figure S5. (a) SEM, (b) HRTEM and (c) STEM and corresponding EDS mapping images of the bare Cu_2O.

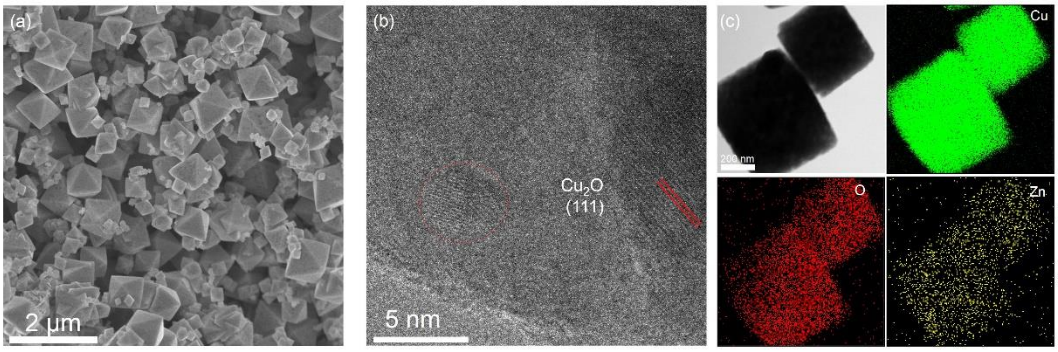

Figure S6. (a) SEM, (b) HRTEM and (c) TEM and corresponding EDS mapping images of Cu_2O-Zn-1%.

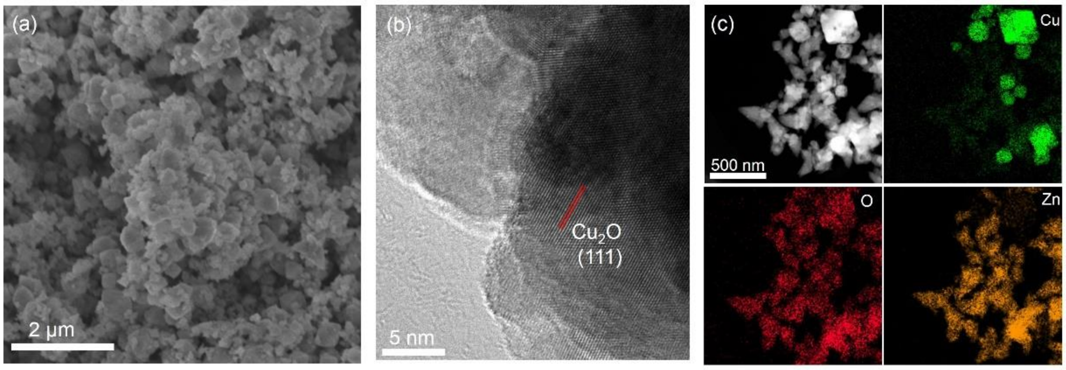

Figure S7. (a) SEM, (b) HRTEM and (c) TEM and corresponding EDS mapping images of Cu_2O-Zn-25%.

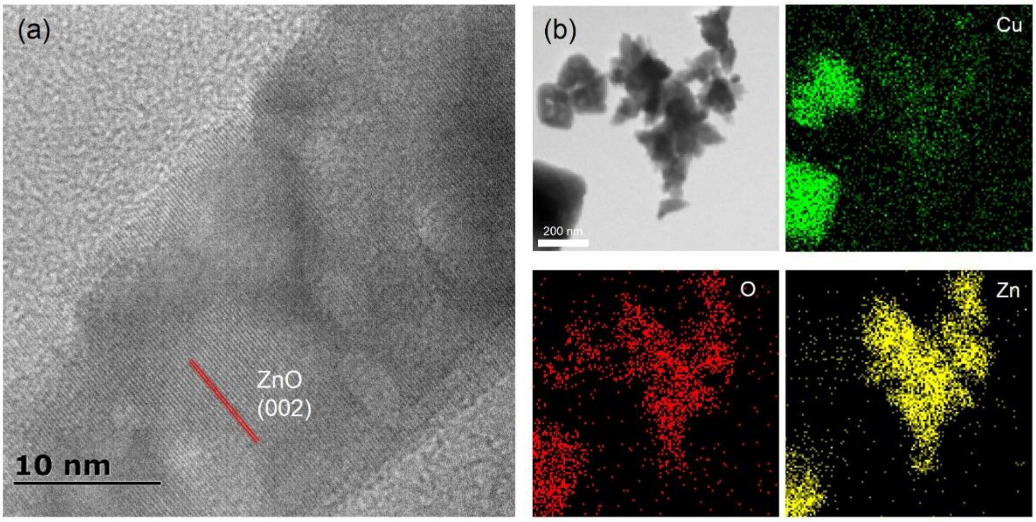

Figure S8. (a) HRTEM, (b) STEM and corresponding EDS mapping images of ZnO nanoparticles in Cu_2O-Zn-25%.

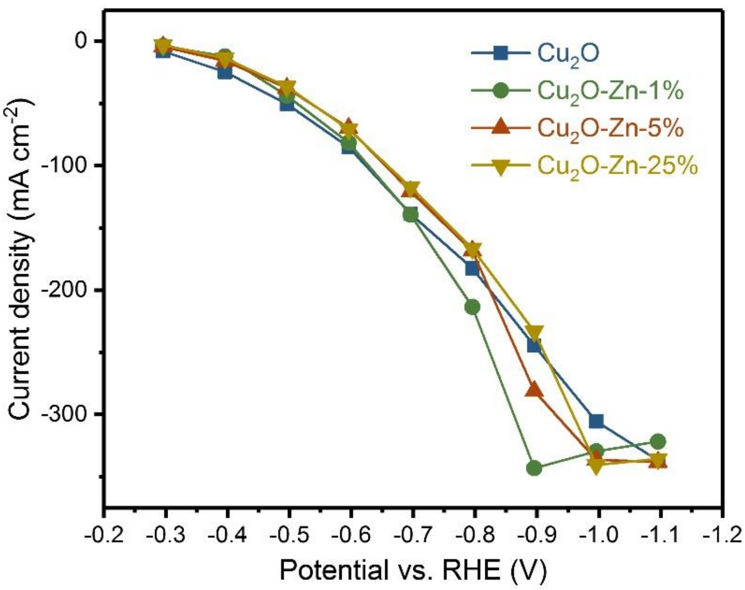

Figure S9. The total current density at different potentials over Cu_2O, Cu_2O-Zn-1%, Cu_2O-Zn-5% and Cu_2O-Zn-25% in a 1 M KOH solution using a commercial gas diffusion electrode-based flow cell.

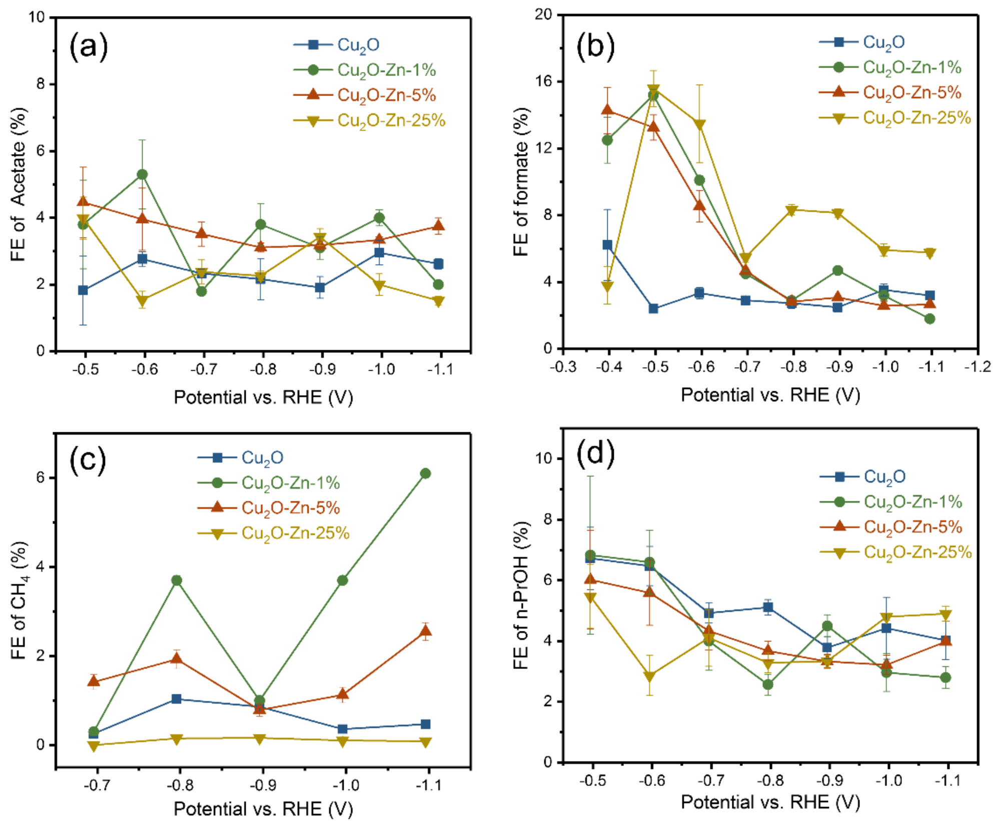

Figure S10. The FEs of (a) acetate, (b) n-PrOH, (c) CH_4 and (d) formate at different potentials over Cu_2O, Cu_2O-Zn-1%, Cu_2O-Zn-5% and Cu_2O-Zn-25% in 1 M KOH solution using a commercial gas diffusion electrode-based flow cell.

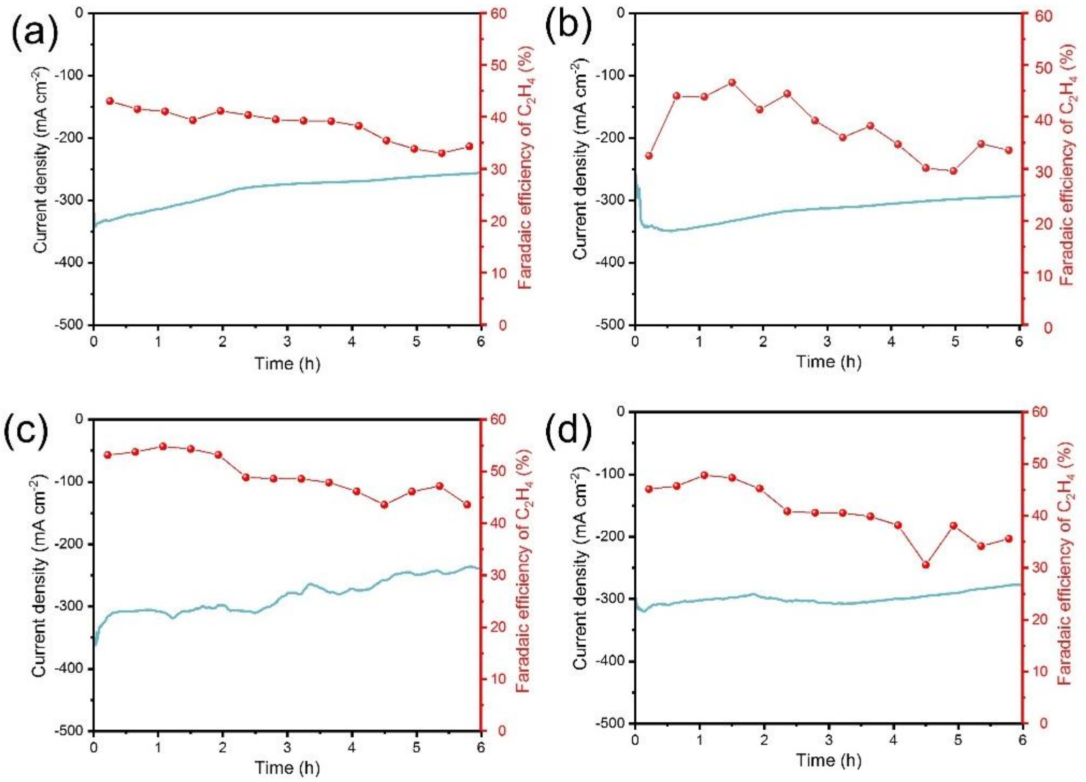

Figure S11. The current density and FE stability of C_2H_4 at –1.0 V over Cu_2O, Cu_2O-Zn-1%, Cu_2O-Zn-5% and Cu_2O-Zn-25% in 1 M KOH solution using a commercial gas diffusion electrode flow cell.

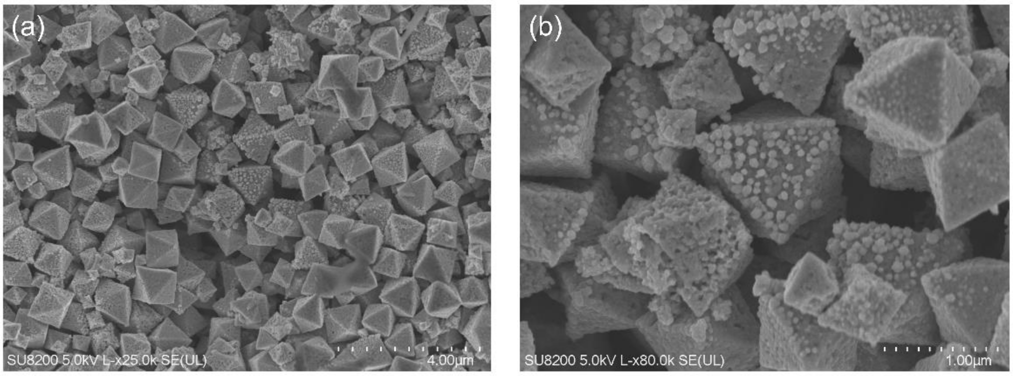

Figure S12. SEM images of Cu_2O-Zn-5% after a long-term electrochemical test.

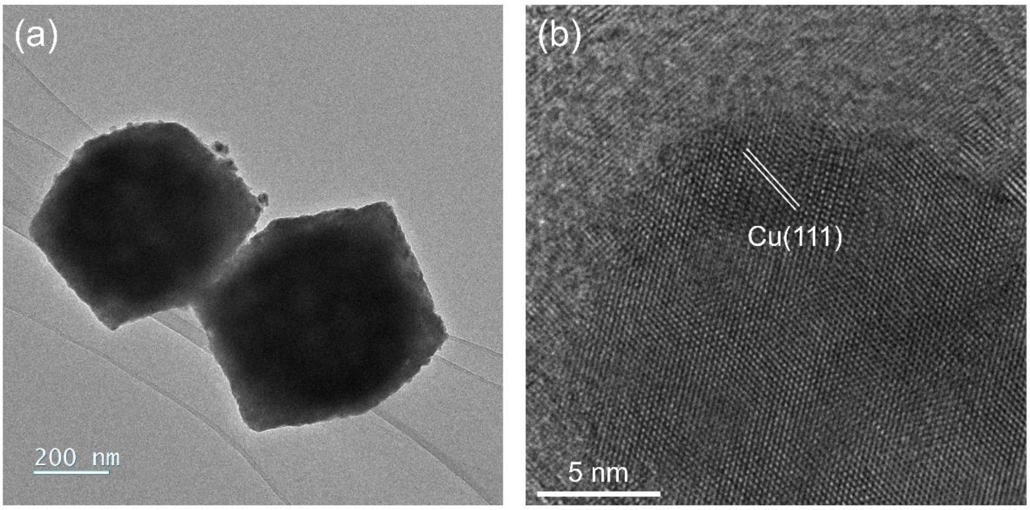

Figure S13. (a) TEM and (b) HRTEM image of Cu_2O-Zn-5% after a long-term electrochemical test.

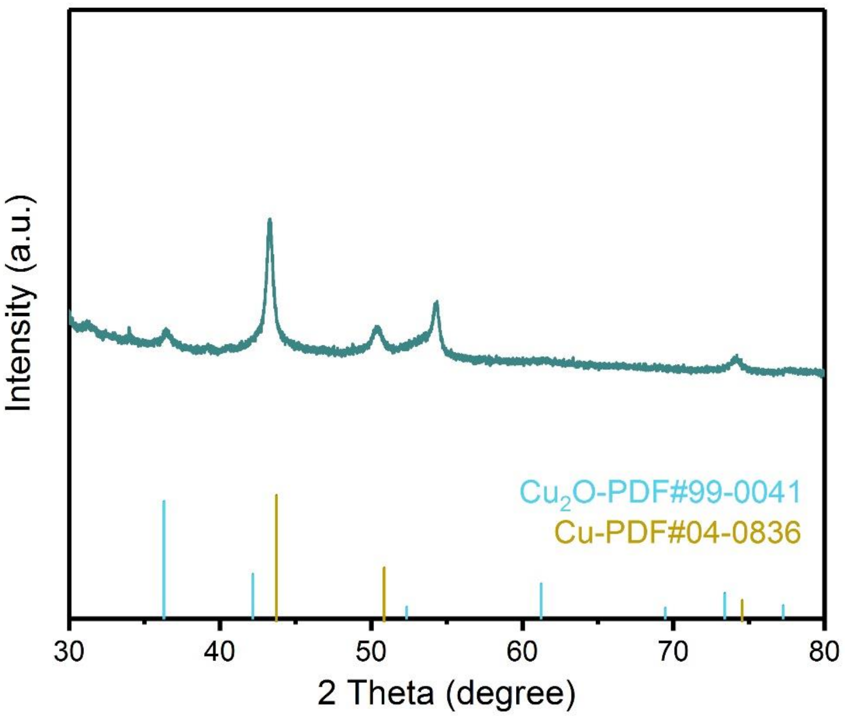

Figure S14. XRD pattern of Cu_2O-Zn-5% after a long-term electrochemical test.

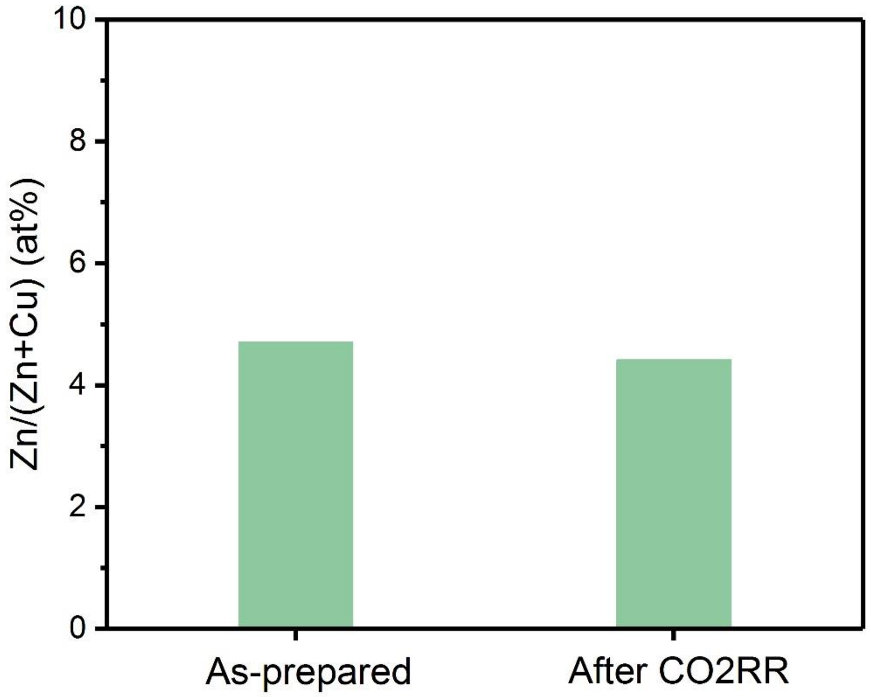

Figure S15. Bulk atomic ratio of Zn and Cu on Cu_2O-Zn-5% catalysts before and after a long-term CO2RR.

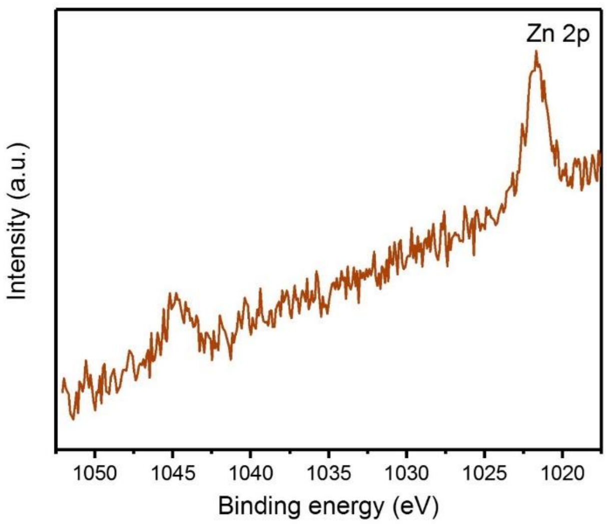

Figure S16. The XPS spectrum of Zn 2p of Cu_2O after 1-hour CO2RR in 0.2 mM Zn^{2+}-added electrolyte at –0.9 V.

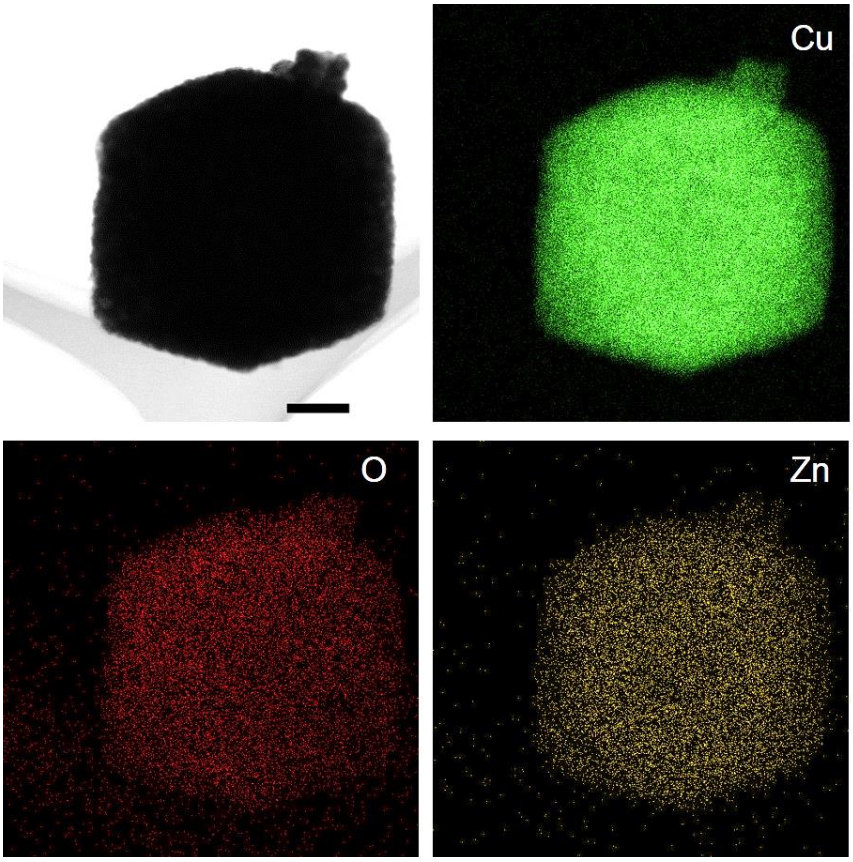

Figure S17. STEM and corresponding EDS element mapping images of Cu_2O after 1-hour CO2RR in 0.2 mM Zn^{2+}-added electrolyte at –0.9 V.

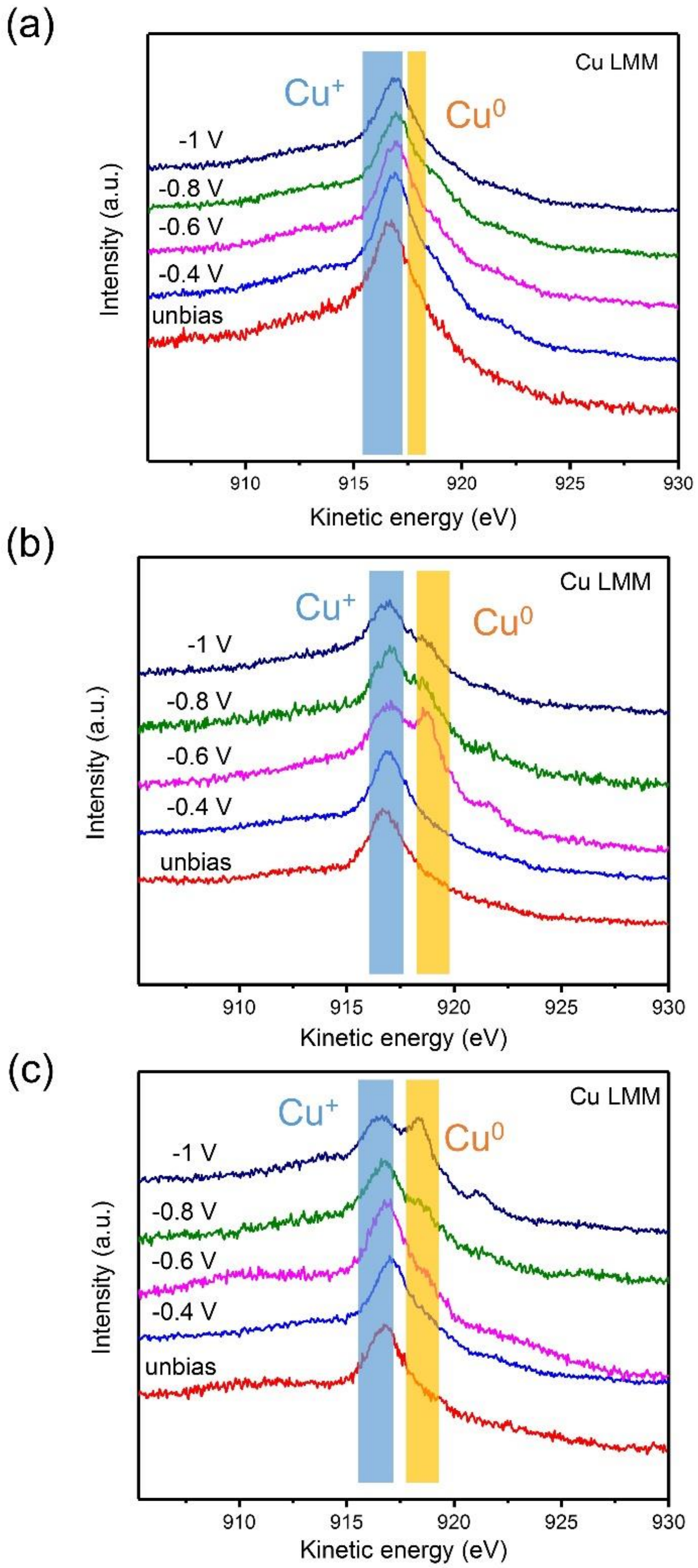

Figure S18. Cu LMM spectra of the bare Cu_2O (a), Cu_2O-Zn-5% (b) and Cu_2O-Zn-25% (c) after CO2RR under different potentials.

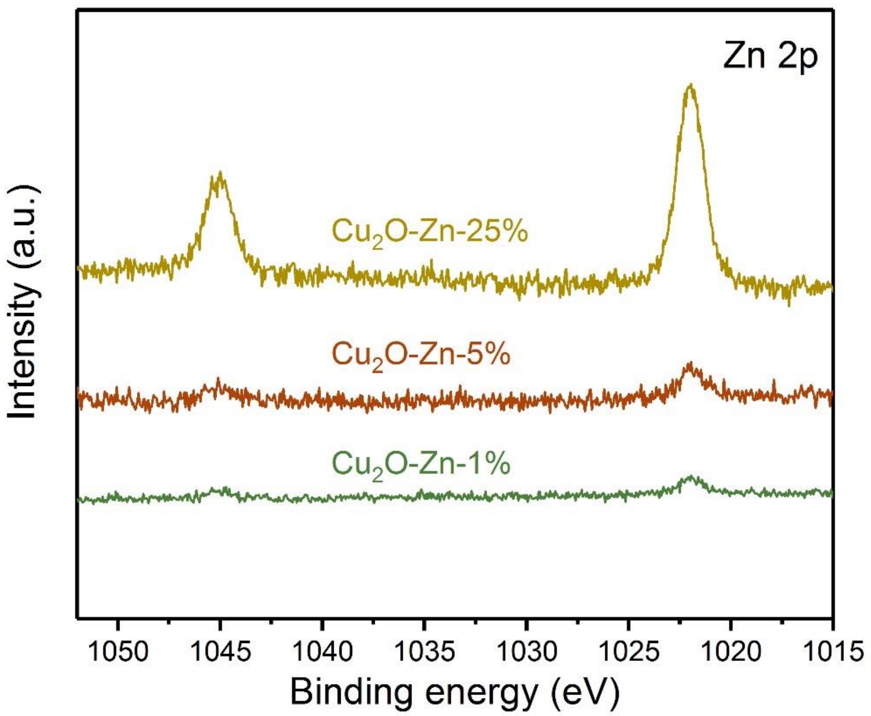

Figure S19. Zn 2p spectra of Cu_2O-Zn-1%, Cu_2O-Zn-5% and Cu_2O-Zn-25% after 1-hour CO2RR at –1.0 V.

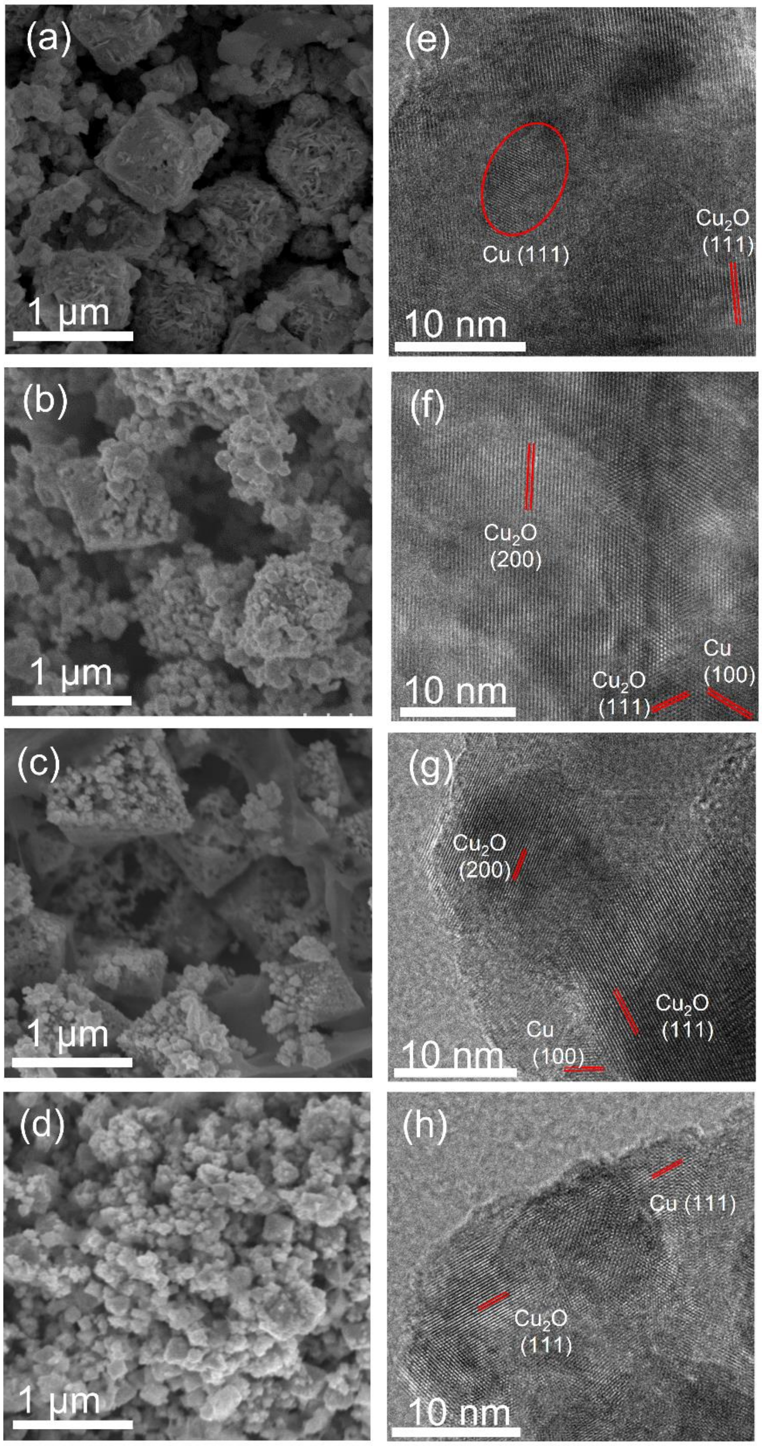

Figure S20. SEM images of (a) Cu_2O, (b) Cu_2O-Zn-1%, (c) Cu_2O-Zn-5% and (d) Cu_2O-Zn-25% after 1-hour CO2RR at –1.0 V. HRTEM images of (e) Cu_2O, (f) Cu_2O-Zn-1%, (g) Cu_2O-Zn-5% and (h) Cu_2O-Zn-25% after 1-hour CO2RR at –1.0 V.

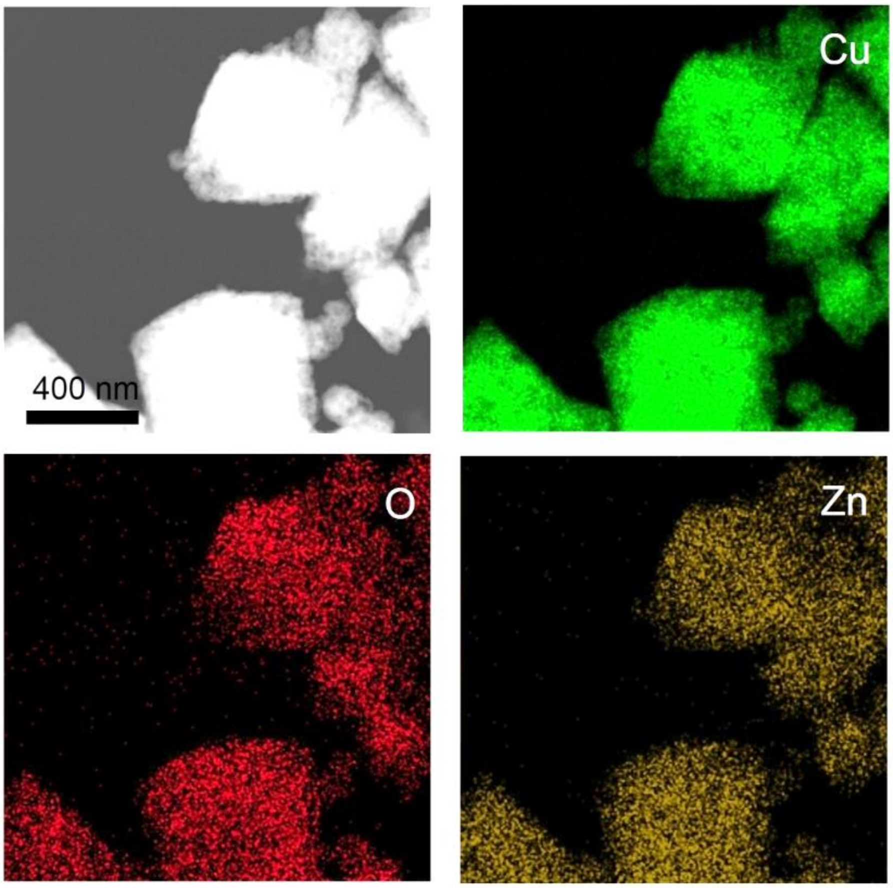

Figure S21. STEM and corresponding EDS element mapping images of Cu_2O-Zn-1% after 1-hour CO2RR at –1.0 V.

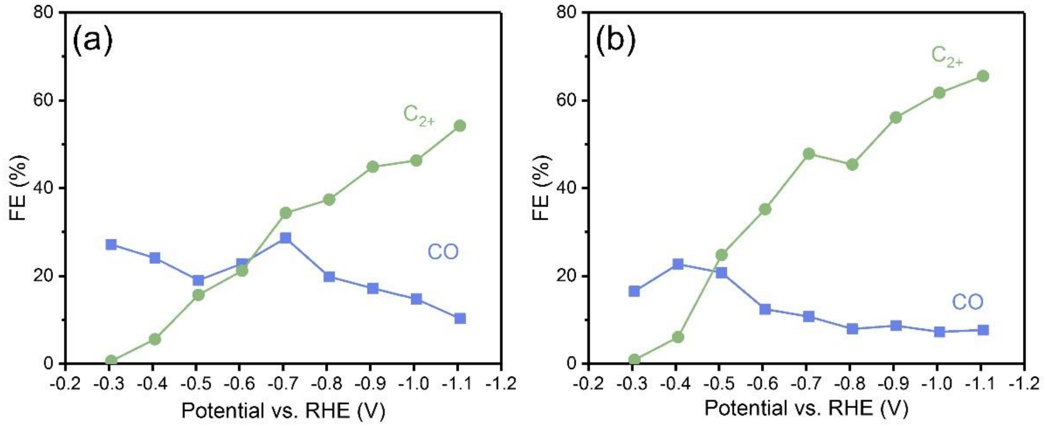

Figure S22. The FEs of CO and C_{2+} products at different potentials over (a) physical-mixed Cu_2O-Zn-5% and (b) dissolution and re-deposition induced physical-mixed Cu-Zn-5% in 1 M KOH solution using a commercial gas diffusion electrode flow cell.

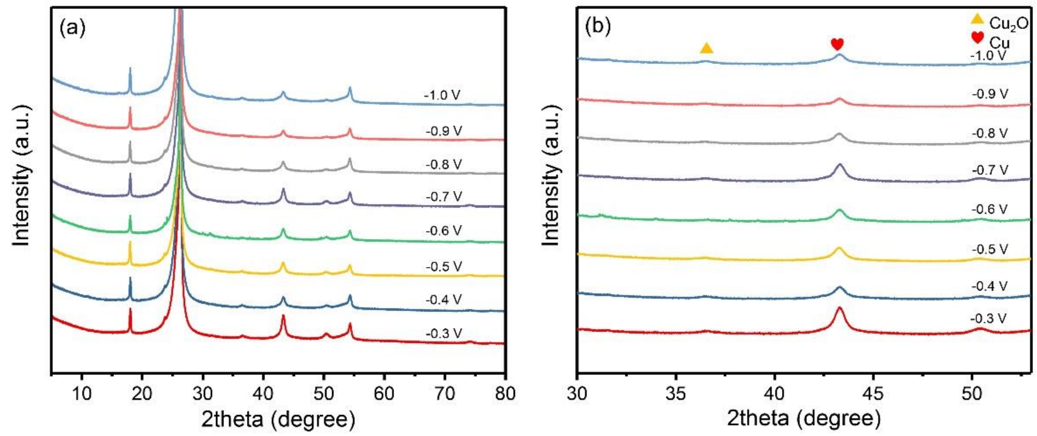

Figure S23. (a) Potential-dependent quasi-operando XRD patterns of Cu_2O-Zn-5% under different potentials for 1-hour CO2RR. (b) Magnified image of Figure S23a.

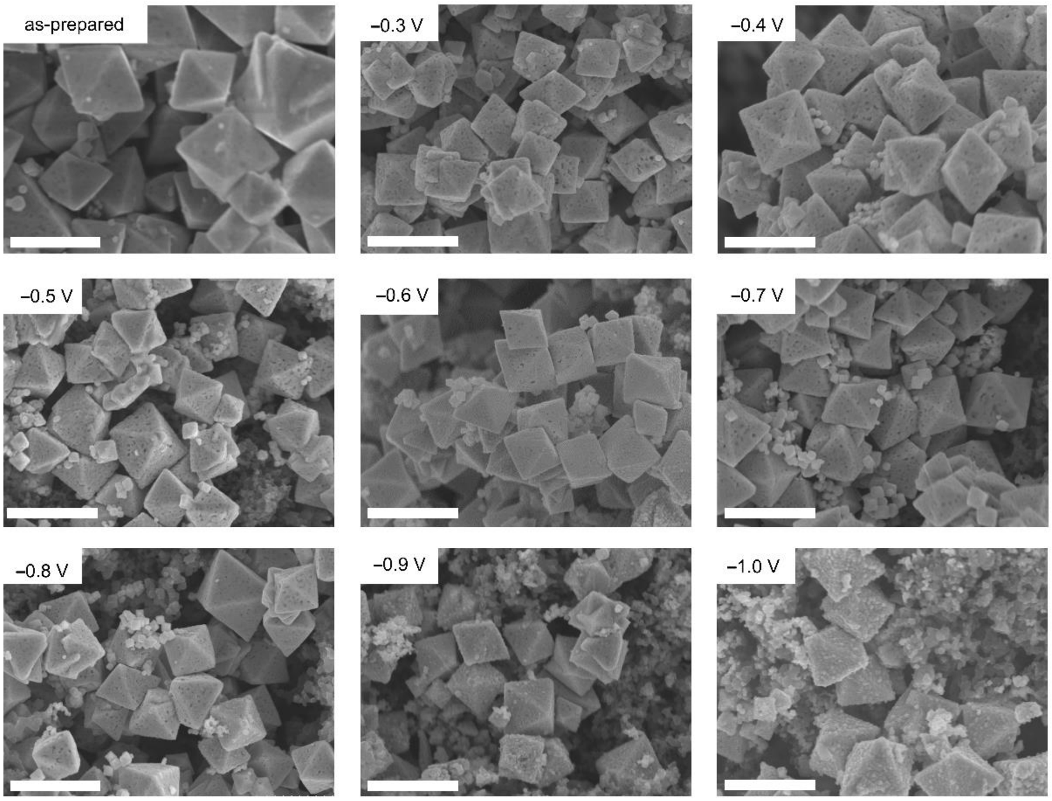

Figure S24. Potential-dependent SEM images of Cu_2O-Zn-5% under different potentials for 1-hour CO2RR. Scale bar: 200 nm.

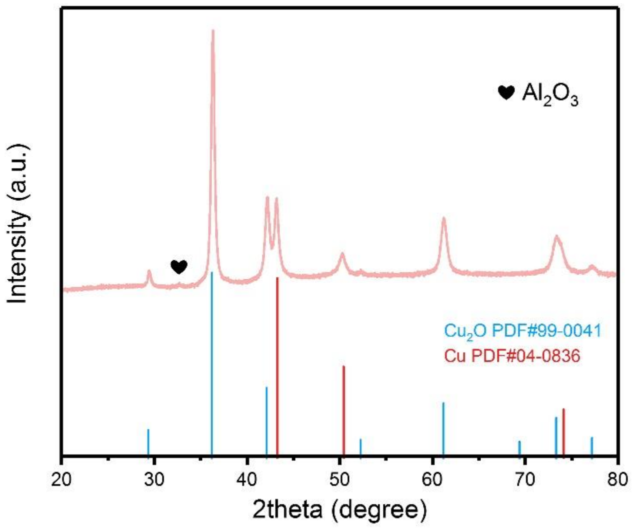

Figure S25. XRD pattern of the Al-doped Cu_2O sample.

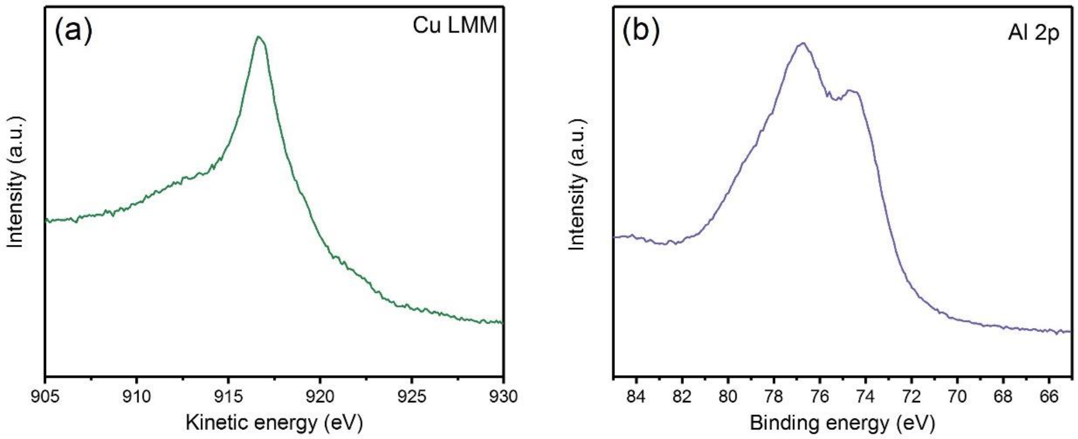

Figure S26. (a) Cu LMM spectrum and (b) XPS of Al 2p in Al-doped Cu_2O.

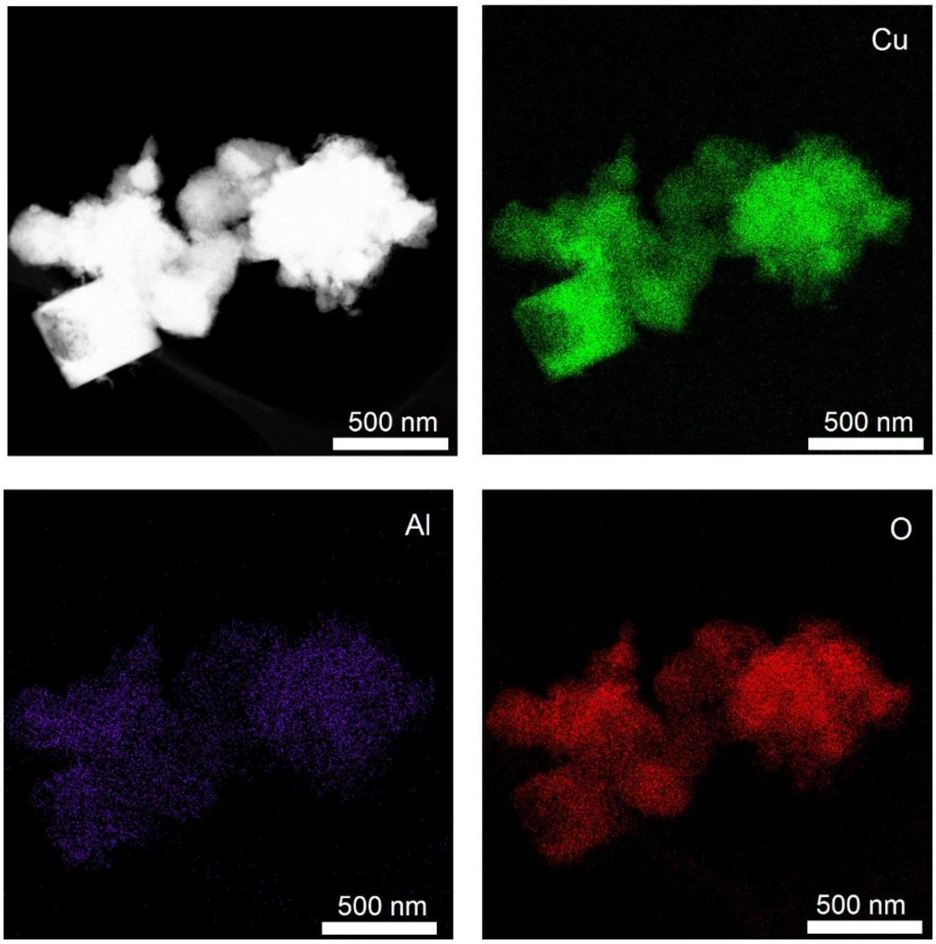

Figure S27. STEM and corresponding EDS-element mapping images of Al-doped Cu_2O.

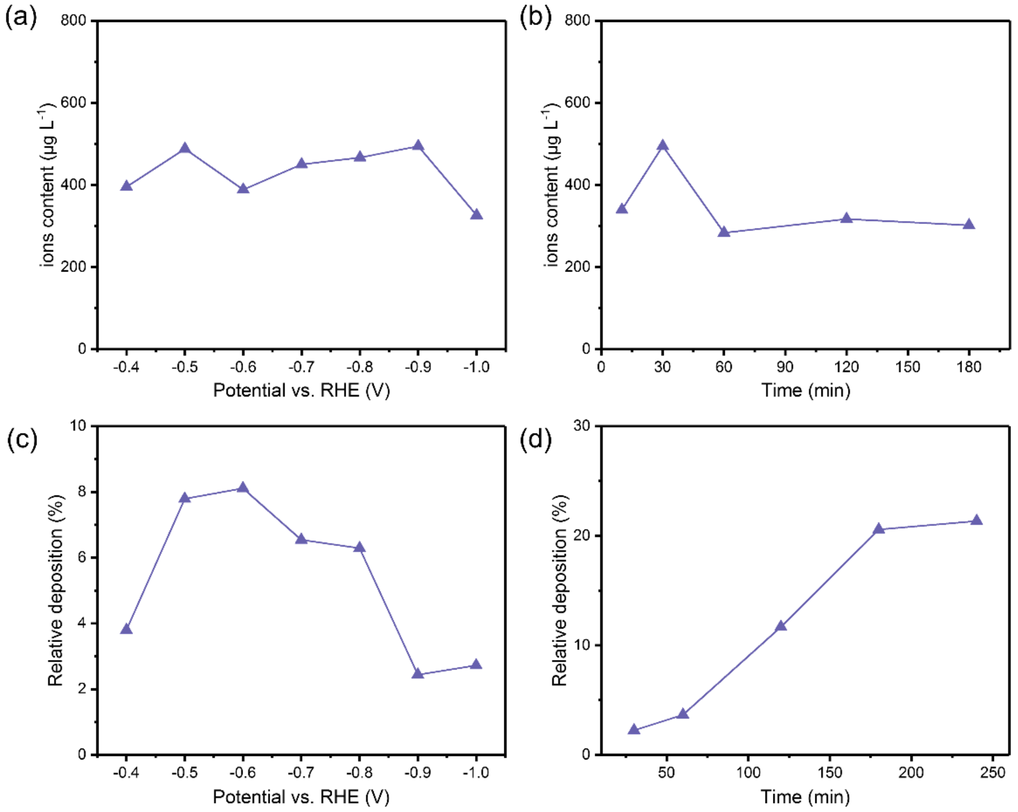

Figure S28. (a) Potential-dependent Al^{3+} concentration in electrolyte using the Cu_2O-Al 5% as catalyst after 30-min CO2RR. (b) Time-dependent Al^{3+} concentration in electrolyte using Cu_2O-Al 5% as the catalyst at –0.9 V. (c) Potential-dependent relative deposition of Al over Cu_2O in 1 M KOH with 0.2 mM Al^{3+} as cathode electrolyte after 30-min CO2RR. (d) Time-dependent relative deposition of Al over Cu_2O in 1 M KOH with 0.2 mM Al^{3+} as cathode electrolyte at –0.9 V. The Al^{3+} concentration (a and b) in electrolyte was obtained by test the electrolyte after CO2RR to confirm the dissolution effect of Al. The relative deposition of Al on the Cu_2O surface was calculated by detecting the reduced amount of Al^{3+} in the electrolyte compared with the initial electrolyte.

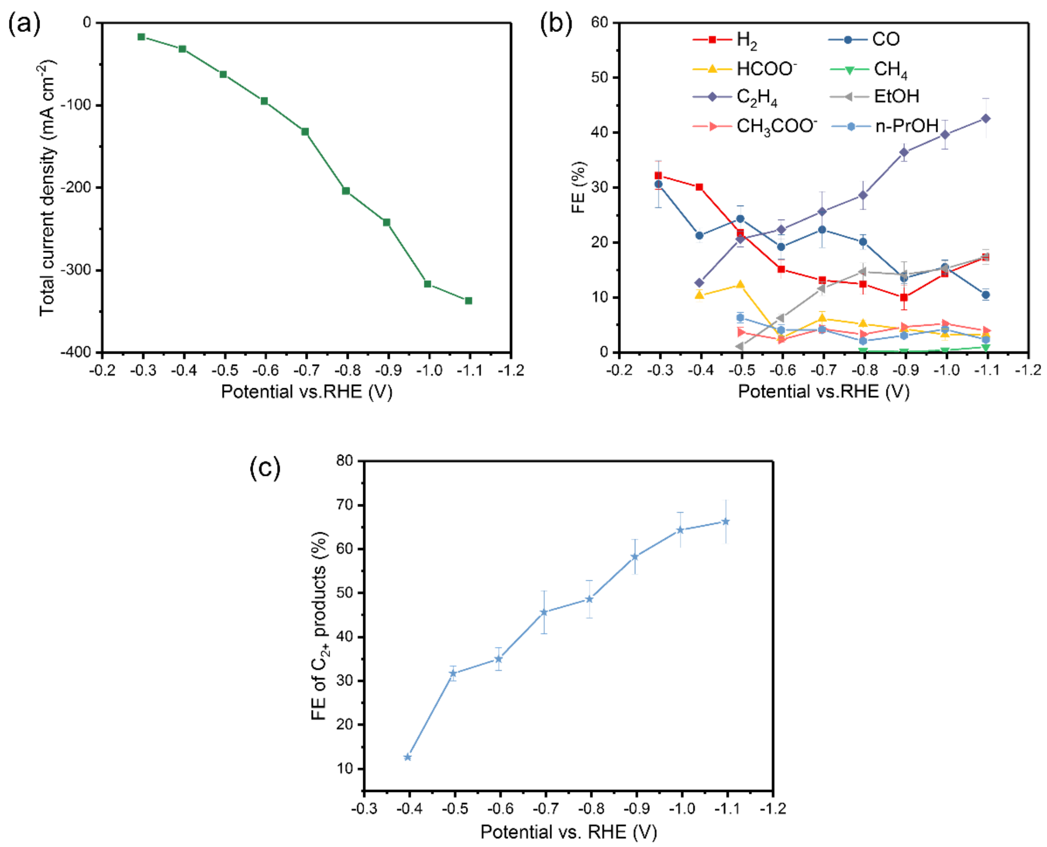

Figure S29. (a) Total current density, (b) FE of different products, and (c) FE of C_{2+} products of Al-doped Cu_2O under different potentials for CO2RR.

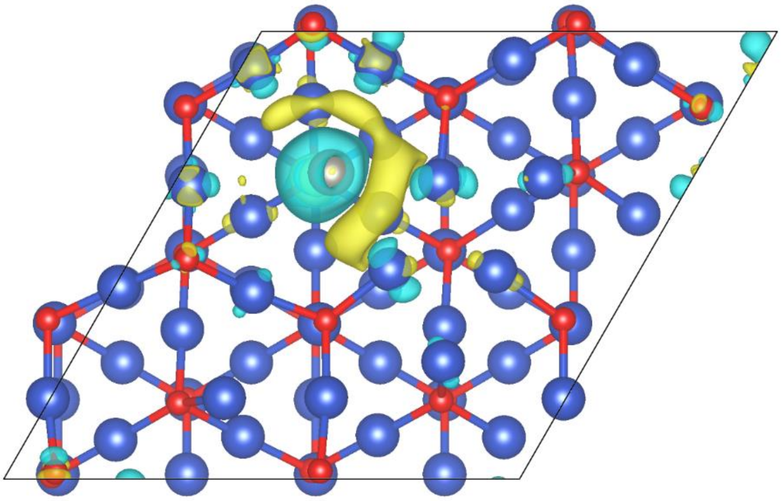

Figure S30. Charge density difference of Zn-doped Cu_2O surface. Yellow and cyan areas indicate charge accumulation and depletion, respectively. The iso-value is 0.002 e/Å^3. Gray, blue, and red balls represent Zn, Cu, and O, respectively.

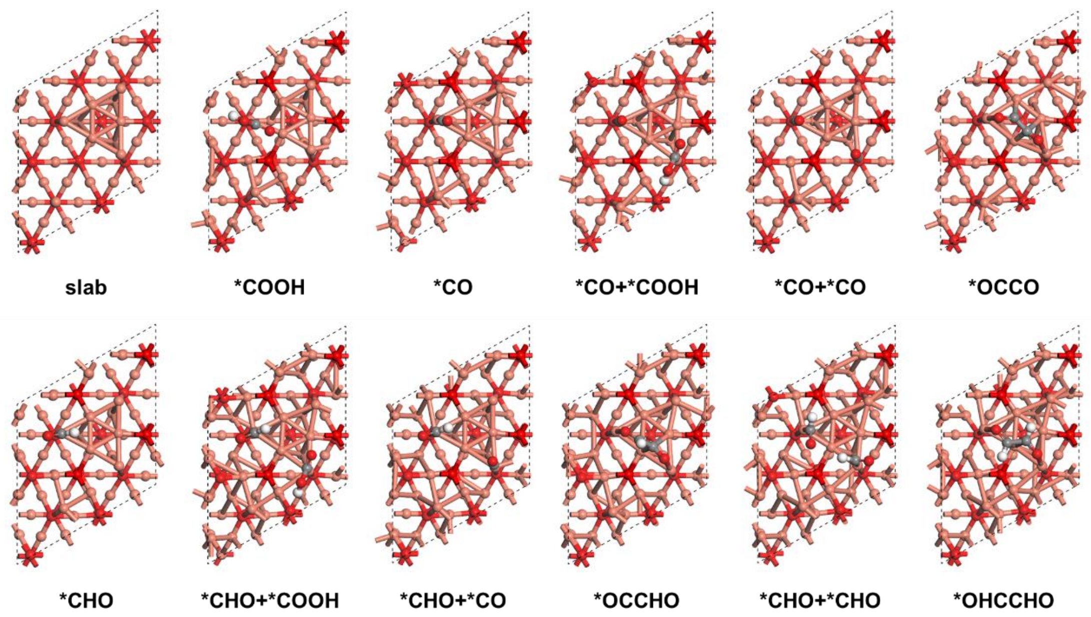

Figure S31. Adsorption configurations of the optimized intermediate structures on Cu_2O (111) surface. Gray, white, orange, and red balls represent C, H, Cu, and O, respectively.

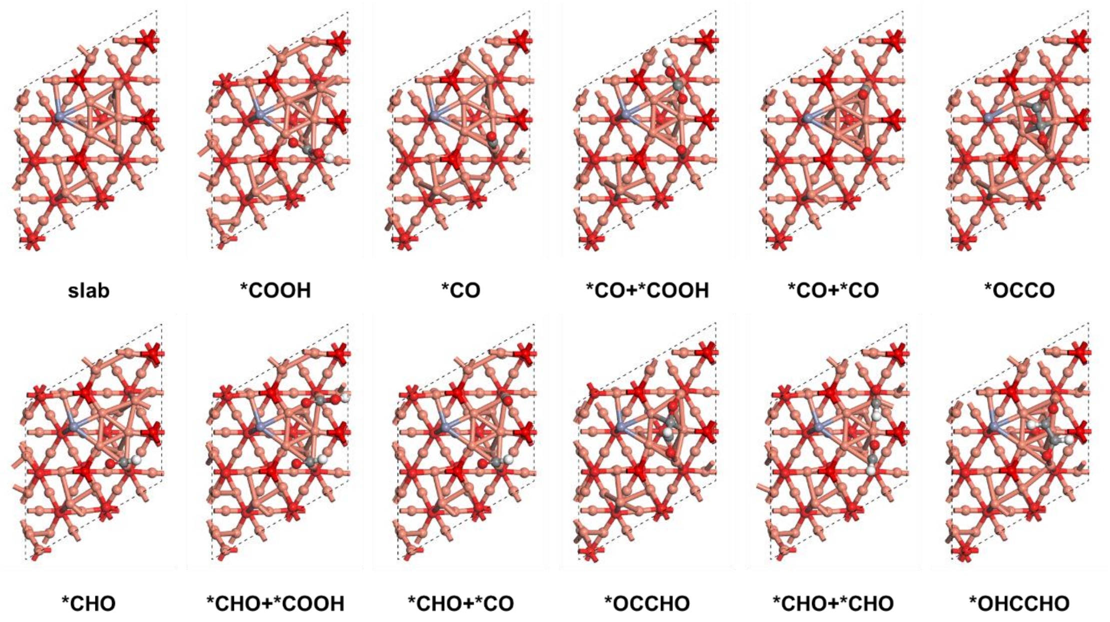

Figure S32. Adsorption configurations of the optimized intermediate structures on Zn-doped Cu_2O (111) surface. Gray, white, violets, orange, and red balls represent C, H, Zn, Cu, and O, respectively.

Table S1. Rate determining steps and corresponding reaction free energies of OC-CO pathway, OC-CHO pathway, and OHC-CHO pathway on Cu_2O and Zn-doped Cu_2O.

Pathway	Cu_2O		Zn-doped Cu_2O	
	ΔG_{max} / eV	RDS	ΔG_{max} / eV	RDS
OC-CO	1.94	*CO + *CO → *OCCO	1.93	*CO + *CO → *OCCO
OC-CHO	1.01	*CO → *CHO	0.52	*CO → *CHO
OHC-CHO	1.01	*CO → *CHO	0.84	*CHO + *CO → *CHO + *CHO

References

[1] a) G. Kresse, J. Furthmüller, *Phys. Rev. B* **1996**, *54*, 11169-11186; b) G. Kresse, D. Joubert, *Phys. Rev. B* **1999**, *59*, 1758-1775.

[2] P. E. Blöchl, *Phys. Rev. B* **1994**, *50*, 17953-17979.

[3] J. P. Perdew, K. Burke, M. Ernzerhof, *Phys. Rev. Lett.* **1996**, *77,* 3865-3868.

[4] a) S. Grimme, J. Antony, S. Ehrlich, H. Krieg, *J. Chem. Phys.* **2010**, *132*, 154104; b) S. Grimme, S. Ehrlich, L. Goerigk, *J. Comput. Chem.* **2011**, *32*, 1456-1465.

[5] A. Werner, H. D. Hochheimer, *Phys. Rev. B* **1982**, *25*, 5929-5934.

[6] a) A. Michaelides, P. Hu, *J. Am. Chem. Soc.* **2001**, *123*, 4235-4242; b) Z.-P. Liu, P. Hu, *J. Am. Chem. Soc.* **2003**, *125*, 1958-1967; c) A. Alavi, P. Hu, T. Deutsch, P. L. Silvestrelli, J. Hutter, *Phys. Rev. Lett.* **1998**, *80,* 3650.
